# New insights into Notch signaling as a crucial pathway of pancreatic cancer stem cell behavior by chrysin-polylactic acid-based nanocomposite

**DOI:** 10.1007/s12672-025-01846-3

**Published:** 2025-02-01

**Authors:** Eman M. Ragab, Doaa M. El Gamal, Fares F. El-najjar, Hager A. Elkomy, Mahmoud A. Ragab, Mariam A. Elantary, Omar M. Basyouni, Sherif M. Moustafa, Shimaa A. EL-Naggar, Abeer S. Elsherbiny

**Affiliations:** 1 Biochemistry Division, Chemistry Department, Faculty of Science, Tanta University, Tanta, 31527 Egypt; 2 Chemistry/Biochemistry Division, chemistry department, Faculty of Science, Tanta University, Tanta, 31527 Egypt; 3 Chemistry/Zoology Division, chemistry department, Faculty of Science, Tanta University, Tanta, 31527 Egypt; 4 Chemistry Department, Faculty of Science, Tanta University, Tanta, 31527 Egypt

**Keywords:** Pancreatic cancer, Cancer stem cells, Notch pathway, Polylactic acid, Chrysin

## Abstract

Pancreatic cancer is an extremely deadly illness for which there are few reliable treatments. Recent research indicates that malignant tumors are highly variable and consist of a tiny subset of unique cancer cells, known as cancer stem cells (CSCs), which are responsible for the beginning and spread of tumors. These cells are typically identified by the expression of specific cell surface markers. A population of pancreatic cancer stem cells with aberrantly active developmental signaling pathways has been identified in recent studies of human pancreatic tumors. Among these Notch signaling pathway has been identified as a key regulator of CSCs self-renewal, making it an attractive target for therapeutic intervention. Chrysin-loaded polylactic acid (PLA) as polymeric nanoparticles systems have been growing interest in using as platforms for improved drug delivery. This review aims to explore innovative strategies for targeted therapy and optimized drug delivery in pancreatic CSCs by manipulating the Notch pathway and leveraging PLA-based drug delivery systems. Furthermore, we will assess the capability of PLA nanoparticles to enhance the bioavailability and effectiveness of gemcitabine in pancreatic cancer cells. The insights gained from this review have the potential to contribute to the development of novel treatment approaches that combine targeted therapy with advanced drug delivery utilizing biodegradable polymeric nanoparticles.

## Introduction

Cancer continues to stand as the foremost contributor to global mortality, placing a significant strain on healthcare resources. Projections for the year 2023 anticipate a total of 1,958,310 newly diagnosed cancer cases and 609,820 cancer-related fatalities in the United States [1]. In the face of the considerable mortality associated with cancer, noteworthy progress has been made in the treatment of individuals afflicted with this disease through extensive decades of research. Key advancements include targeted therapy, immunotherapy, and the integration of multiple therapeutic approaches, collectively referred to as combinational therapy [2].

Pancreatic cancer (PC) is a lethal ailment, persisting as one of the most formidable malignancies to manage due to its aggressive characteristics and constrained therapeutic alternatives. Despite substantial advancements in the domain of cancer therapeutics, the five-year survival rate for individuals with pancreatic cancer remains below 10%, underscoring the imperative requirement for novel and inventive therapeutic approaches [3].

**Fig. 1:**
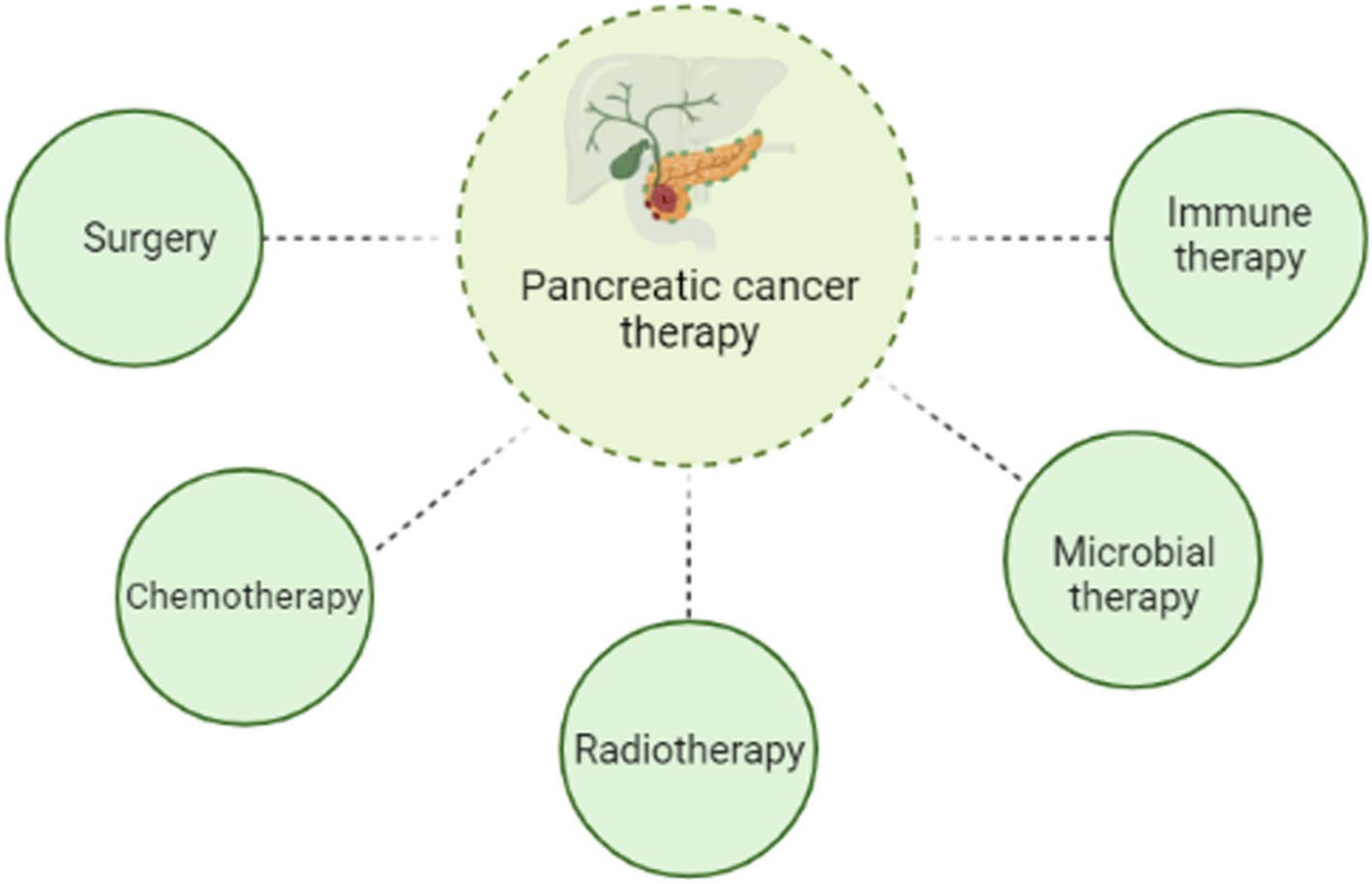
Schematic of different methods for PC treatment

The primary therapeutic modalities available to patients diagnosed with pancreatic cancer encompass a range of interventions, influenced by the specific cancer subtype, disease stage, and additional variables. These treatment options comprise surgical procedures, chemotherapy, radiation therapy, immune-based therapies, and microbial-based therapies (Fig. 1) [4]. The existing gold standard treatment for individuals with resectable pancreatic ductal adenocarcinoma (PDAC) is surgery with the intent of achieving a cure. However, a substantial majority of PDAC patients are ineligible for surgical intervention due to progression into an advanced stage or the emergence of distant metastases [5]. Furthermore, individuals diagnosed with PDAC may encounter instances of both local and systemic recurrence following surgical intervention [6].

Radiation therapy employs elevated-energy X-rays to provide therapeutic intervention to specific PDAC patients, either as an adjuvant approach subsequent to surgery or as a neoadjuvant treatment in conjunction with chemotherapy or immunotherapy [6]. Immune checkpoint blockade (ICB) therapy has gained approval for a range of cancer types, including melanoma, lung cancer, renal cell carcinoma, and head and neck squamous cell carcinoma [7]. Nonetheless, PC is perceived as exhibiting lower immunogenicity in comparison to other malignancies [8].

Microbial therapy plays a pivotal role in the modulation of cancer progression and the subsequent response to cancer treatment interventions [8]. The researchers found that the macrophages contained in the patient-derived bacterial extracts reduced the activation of CD4 + (helper) and CD8 + T (helper or killer) cells after transplanting intestinal bacterial extracts from PC patients into a mouse model.This suppression of antigen presentation by macrophages led to heightened activation of distinct pattern recognition receptors (PRRs) within the tumor macrophages. Conversely, when pancreatic tumors grew within hosts subjected to antibiotic ablation, markedly contrasting outcomes were observed [9]. To date, the impact of the gut microbiota on generating systemic immunity and tumor-specific immunity in PC has been validated, yet the precise underlying mechanism requires further investigation [8]. However, the current cancer therapies are not without their associated adverse effects, and the atypical extracellular matrix (ECM) present in solid tumors creates a formidable barrier to the infiltration of therapeutic agents or immune cells. Consequently, recent endeavors have been directed towards the formulation of targeted anticancer medications, aimed at mitigating these constraints.

Focusing on cancer stem cells (CSCs) presents a prospective approach for PC treatment. CSCs are thought to hold a pivotal position in the advancement and advancement of PC, owing to their capacity for self-renewal and differentiation across diverse cell lineages, thus facilitating tumor proliferation and metastasis. The Notch signaling pathway is acknowledged for its role in governing CSC self-renewal, differentiation, and programmed cell death, rendering it an appealing target for therapeutic intervention in the context of cancer [10].

Flavonoids offer several beneficial bioactivities, such as anticancer, anti-inflammatory, and antioxidant properties. Dietary supplements containing flavonoids are currently being extensively investigated in clinical studies for the prevention and/or treatment of a variety of illnesses [11]**.** However, flavonoids' limited bioavailability and bioactivity, which are likely caused by their poor water solubility, high metabolism, and low systemic absorption, significantly hinder their therapeutic use. Therefore, a possible strategy for getting around these problems is to formulate flavonoids into innovative delivery methods [12]. As science and technology have advanced over the past few decades, more and more innovative delivery systems have been created to enhance the safety, effectiveness, and patient compliance of bioactive substances. These systems primarily target delivery, enhance aqueous solubility, improve dissolution behavior, prevent undesirable metabolism, and optimize administration routes. Thus, it is generally accepted that incorporating flavonoids into innovative delivery systems is a viable strategy to boost their bioavailability and bioactivity. For the delivery of flavonoids, a number of innovative delivery systems have been developed, such as complexes, emulsions, solid dispersions, crystal engineering preparations, immunosensors based on nanomaterials and different nanocarriers that treat a pleathora of cancer types like prostatic, breast, pancreatic cancer [12–14]. Neoplastic illness is still a serious public health concern, and nanotechnologies offer potential methods for its diagnosis and therapy. However, it can be difficult to distribute the therapeutic agent effectively, due to common characteristic of many cancer-related deaths is tumor metastasis [15, 16]**.**

Polymeric nanoparticles have garnered significant interest in recent years as effective vehicles for drug delivery. These systems merge established drug delivery techniques with engineered technologies, enabling precise targeting of drug release sites within the body and controlling the release rate. The utilization of biodegradable and bio-absorbable polymers, particularly hydrogels like poly(lactic acid) and poly(glycolic acid) and their copolymers, serves as a key approach for constructing these advanced drug delivery systems [17, 18]. The use of biodegradable and bio-absorbable polymers provides a safe foundation for delivering medicine without harming the body, regardless of whether the drug delivery system uses a biodegradable implant to distribute medication subcutaneously or deep within the body.

The most prevalent types of macromolecules include biopolymers, which encompass nucleic acids, proteins, carbohydrates, lipids, as well as large non-polymeric entities such as lipids and macrocycles [19]. Synthetic macromolecules encompass various materials, including plastics, synthetic fibers, and innovative substances like carbon nanotubes [20]. Moreover, the recurring building blocks of nucleic acids, saccharides, or amino acids, the molecular structures may incorporate diverse chemical side chains that provide to the molecular functions.

Polylactic acid (PLA) and polyhydroxyalkanoates (PHAs) stand as representative biopolymers occurring in microorganisms or genetically engineered organisms using conventional chemical methods. These involve polysaccharides sourced from cellulose and proteins derived from collagen or milk.

This review aims to introduce and analyze innovative strategies for targeted therapy and drug delivery in PC stem cells by modulating the Notch signaling pathway and enhancing drug release through the utilization of PLA-based systems. Specifically, the investigation will assess the effectiveness of chrysin-encapsulated PLA nanoparticles in targeting pancreatic CSCs and modulating the Notch pathway. Additionally, the study will evaluate the capacity of these nanoparticles to improve the bioavailability and efficacy of gemcitabine in pancreatic cancer cells.

## Stem cells differentiation

**Fig. 2:**
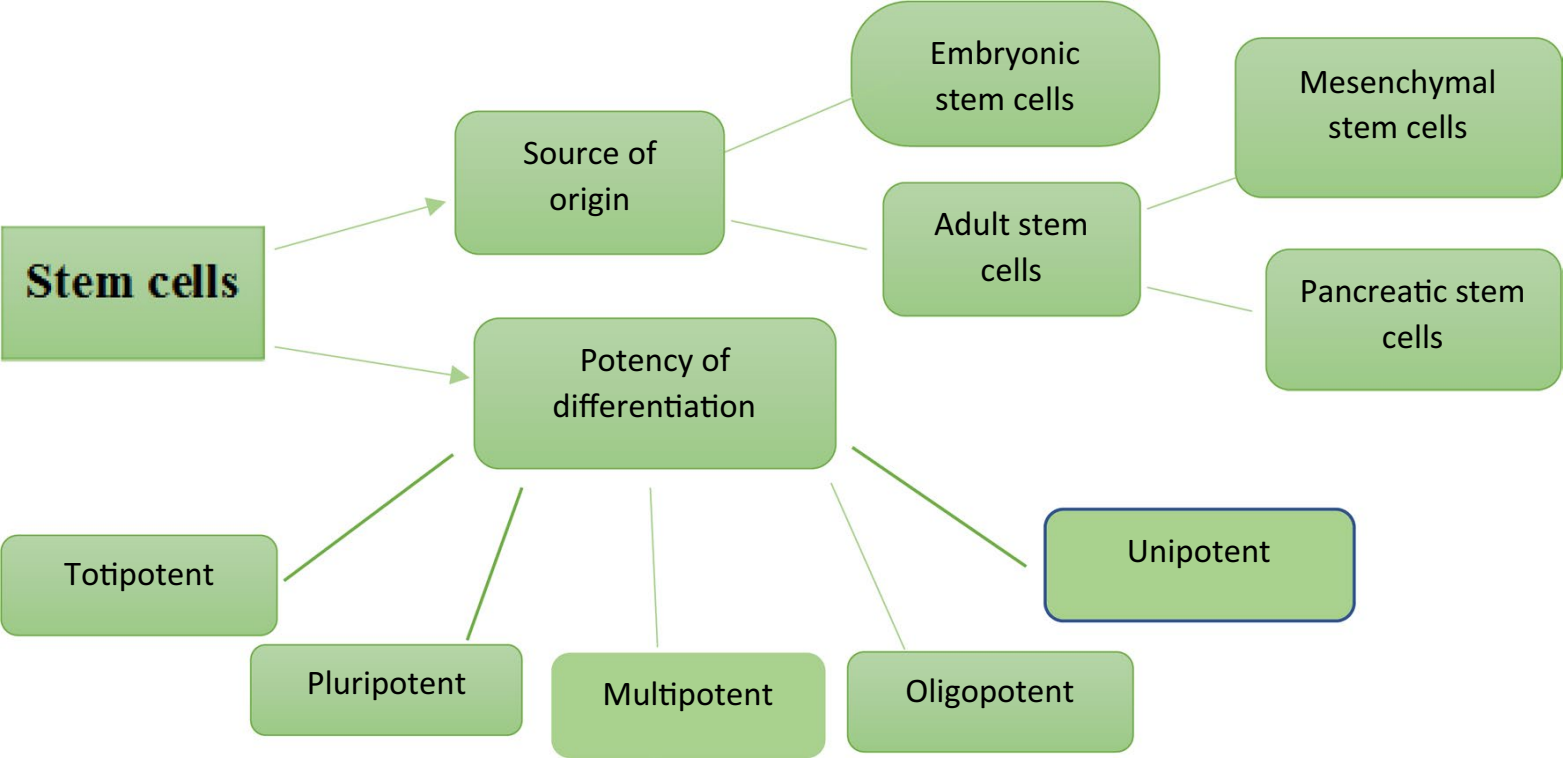
Schematic of the classification of stem cells

The human body harbors undifferentiated stem cells that possess a defining characteristic: their unlimited potential for proliferation. This attribute enables self-renewal and the ability to differentiate into diverse lineages, beginning from embryonic stem cell development (ESC) and continuing into adulthood through adult or somatic stem cells. This regenerative capacity might extend to CSCs and induced pluripotent stem (IPS) cells. Self-renewal results in the generation of additional undifferentiated stem cells, while differentiation leads to the formation of mature cell types. During embryonic development, embryonic stem cells can give rise to all organ types. In adulthood, adult stem cells play a critical task in the replacement and repair of adult tissues [21]**.** Stem cells can be grouped in line with their source of origin and potency of differentiation, as shown in Fig. 2.

## Cancer stem cells (CSCs)

Despite the wealth of research and improvements in cancer therapy, cancer continues to be one of the leading causes of death worldwide [22]. CSCs, alternatively told as tumor-initiating cells (TICs), constitute a distinct subset within the tumor cell population, often referred to as "cancer root cells." These cells possess the capability for self-renewal, giving rise to diverse malignant stem cells and tumor cells that collectively constitute the tumor bulk. Despite their minimal representation (0.05–3% of total cancer cells) within the overall tumor mass, the distinctive cellular attributes of CSCs designate them as the foremost impediment in achieving effective tumor treatment [23].

As per the CSC concept, tumors exhibit a linear cellular hierarchy with CSCs occupying the apex. These cells are responsible for sustaining tumorigenic properties and reproducing the diverse cellular composition inherent in the primary tumor [24].

The production of reactive oxygen species (ROS) is suppressed by the redox system, while drug-resistant transporters are extensively expressed in both CSCs and conventional cancer cells, as well as in normal stem cells. However, in contrast to regular stem cells, CSCs possess a unique metabolic adaptability enabling them to switch between oxidative phosphorylation (OXPHOS) and glycolysis as their predominant energy generation pathways. The defining feature setting CSCs apart from typical cancer and normal cells is their predominantly stem-like characteristics [23].

The diversification within cancer has been elucidated through two models: the hierarchical model of cancer stem cells and the stochastic model of clonal evolution [25]. One of the primary issues with treatment is the heterogeneity of CSCs since these cells help cancer cells survive under difficult circumstances and are resistant to therapy [26, 27]. Because of this, the survival of tumor cells and their metastasis into other tissues are increased by the heterogeneity of CSCs [28, 29]. The CSC model garnered significant attention upon the emergence of evidence substantiating its applicability in human cases of acute myeloid leukemia (AML) and breast cancer [30]. The discovery involved the identification of a minor subset of cancer cells demonstrating the ability to initiate leukemia upon transplantation into immunocompromised mice. However, compelling evidence supporting the existence of CSCs is lacking in a range of solid tumor types including brain [31, 32], prostate [33, 34], colon [35, 36], pancreatic [37], ovarian [38], and lung [39]. The CSC model has garnered significant interest due to its ability to elucidate the clinical observation that despite the initial appearance of cancer cell elimination by many treatments, the disease often experiences recurrence. Consequently, the targeting of CSCs within the tumor becomes a critical imperative in the clinical setting to prevent tumor relapse [30].

Genetic and epigenetic alterations impacting crucial genes associated with cancer cell viability, proliferation, and metastasis intricately govern tumor plasticity. An auspicious avenue for achieving comprehensive cancer eradication lies in the exploration of epigenetic regulators associated with the survival of CSCs, presenting a promising treatment strategy [40]. The diversity in both genetic and epigenetic characteristics within the CSC subset significantly contributes to the intratumoral heterogeneity observed. Moreover, the self-renewal proficiency inherent in the CSC subpopulation plays a pivotal role in instigating tumor relapse [41]. Except for a few specific cases, the majority of CSCs exhibit pronounced resistance to chemotherapy and radiation interventions due to an array of factors. These include a conducive tumor microenvironment, heightened expression of drug efflux pumps, the presence of pro-survival and anti-apoptotic signals, intracellular drug-inactivating enzymes, and enhanced DNA repair mechanisms [42]. DNA repair enzymes, notably Excision Repair Cross-Complementation Group 1 (ERCC1) and O(6)-methylguanine-DNA methyltransferase, have been noted to exhibit elevated levels in CSCs compared to non-CSCs [43, 44]. CSCs display heightened levels of expression for DNA damage checkpoint response molecules, including Nijmegen breakage syndrome protein 1 (NBS1), Checkpoint kinase 1 (Chk1), and Checkpoint kinase 2 (Chk2). These molecules play a pivotal task in the repair of DNA destruction caused by therapeutic agents [22].

**Fig. 3:**
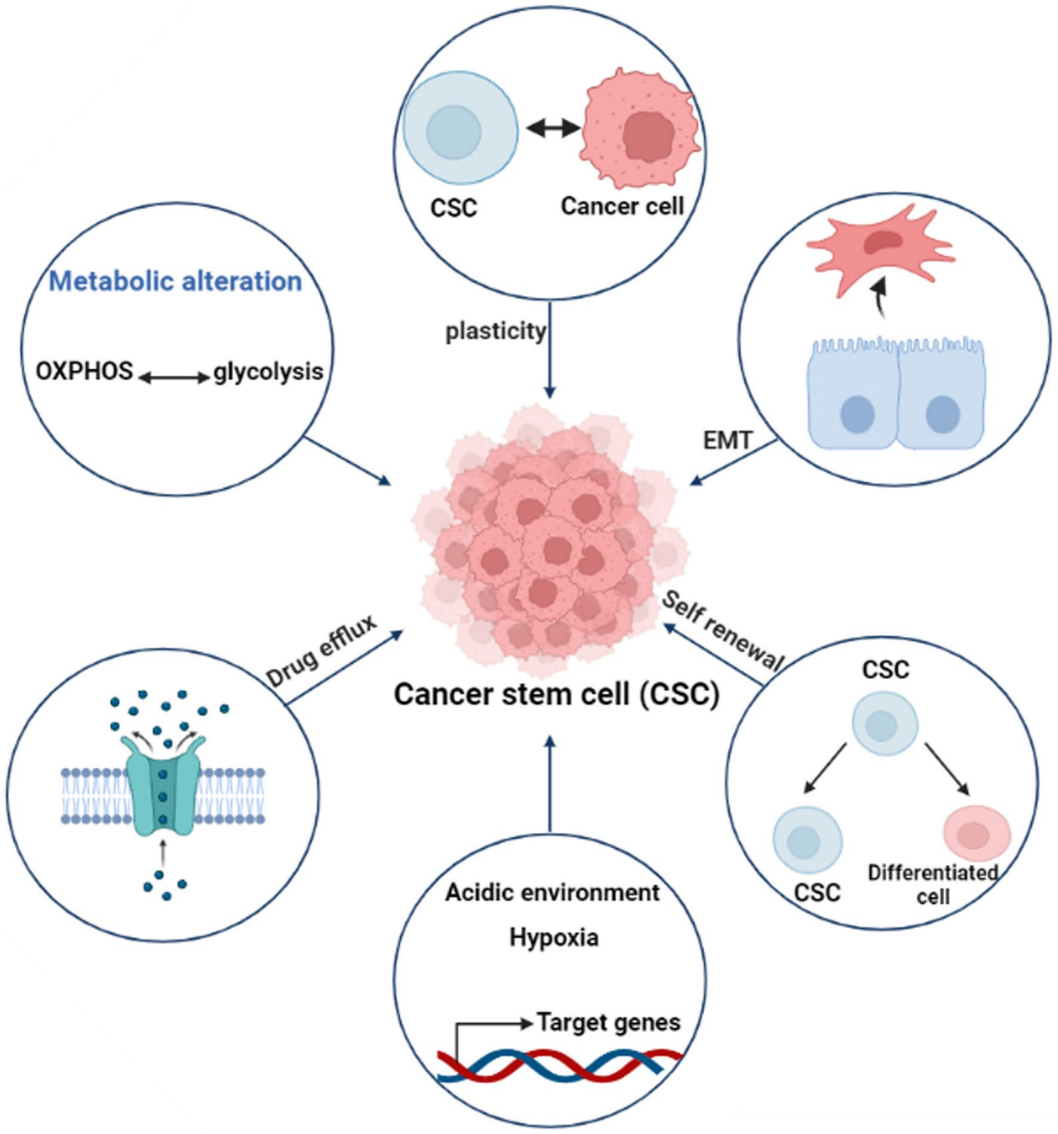
Schematic of the unique feature of CSCs

CSCs possess a robust ability to efflux drugs, rendering them resistant to anticancer treatments. A favorable microenvironment for CSCs is characterized by an acidic pH (ranging from 6.8 to 7) and a hypoxic state with oxygen tension below 5% [45]. This specific microenvironment can trigger changes in gene expression, resulting in heightened angiogenesis, stem cell properties (stemness), and the upregulation of drug efflux transporters. Furthermore, CSCs undergo a genetic process that involves the alteration of the epithelial-to-mesenchymal transition (EMT), a transformation crucial for their readiness to migrate to adjacent tissues **(**Fig. 3**) **[23]. Subsequently, during the mesenchymal to epithelial transition (MET), these cells re-establish attachment to the basement membrane matrix, leading to the formation and growth of tumors. Another distinctive attribute of CSCs is their ability to switch between oxidative and glycolytic metabolism based on the intracellular oxygen levels **(**Fig. 3**).** Furthermore, CSCs possess elevated levels of antioxidant molecules, such as glutathione (GSH), which have a crucial role in sustaining and preserving stemness, proliferation, survival, and contribute to maintaining cellular homeostasis [46, 47].

## Pancreatic cancer stem cells

In 2020, about 0.5 million people received a diagnosis of pancreatic cancer, a serious health issue that affects people all around the world. In 2023, about 64,000 adults in the US will receive a PC diagnosis [48]**.** Pancreatic cancer is among the deadliest cancers worldwide, it is currently the third leading cause of death from cancer [49]. The advanced stage diagnosis, which causes the cancer to spread quickly to the circulatory system and other distant organs, is mostly to blame for this incredibly bad prognosis. Genetic, lifestyle, and environmental factors are among the many and varied risk factors for pancreatic cancer. Genetic factors are thought to be responsible for 10% of pancreatic cancer fatalities, while lifestyle variables account for the remaining 40% [49]**.** It is conceivable that distinct CSC signatures may be associated with the recurrence of PC and the progressionk of the disease [50]**.** Gaining a comprehensive comprehension of the non-genetic determinants contributing to treatment resistance and the recurrence of tumors in PC necessitates a rigorous investigation into the heterogeneity of CSCs, encompassing their biological characteristics and functional attributes.

A minute fraction of the cancer cells within the primary tumor holds the capability to engage in Epithelial-to-Mesenchymal Transition (EMT), a process crucial for initiating tumor invasion and facilitating metastatic dissemination [51, 52] in human cancer patients, involving pancreatic ductal adenocarcinoma (PDAC) [53–55]. Moreover in PCSCs and circulating tumor cells [53, 55]. In individuals with cancer, the process of metastatic seeding often initiates prior to the detection of the main tumor, and dispersed tumor cells may keep on quiescent at secondary places before eventually giving rise to metastatic tumors. A significant proportion of mortality associated with PC is attributed to the presence of metastatic disease.

## Epithelial-mesenchymal transition (EMT)

Mesenchymal stem cells (MSCs) represent a type of stromal cell with the capacity for multilineage differentiation and the capability to undergo self-renewal. MSCs can be obtained from various tissue sources, including adipose tissue, bone marrow, menstrual blood, endometrial polyps, and the umbilical cord. These resources are particularly advantageous for tentative and possible clinical uses due to their ease of extraction and the substantial yield obtained. Under specific in vitro conditions, MSCs can differentiate into diverse lineages of cells originating from the mesoderm, ectoderm, and endoderm. These include cells such as bone, adipose tissue, chondrocytes, muscle cells, neurons, islet cells, and liver cells. The process of differentiation is regulated by genetic mechanisms, primarily involving transcription factors. Certain regulatory genes direct progenitor cells towards a specific lineage, governing differentiation along a particular phenotypic pathway. To optimize proliferation and differentiation, a tailored microenvironment can be established using biomaterial scaffolds. This environment provides the ideal conditions for MSCs by incorporating growth factors and induction chemicals, facilitating their development along desired pathways.

The inherent inability of normal epithelial cells to migrate is attributed to the presence of structural intracellular junctions, which encompass adherent junctions, tight junctions, and gap junctions [56]. Intracellular junctions are perceived as potential impediments to epithelial cell invasion, metastasis, and migration. To attain migratory capabilities, epithelial cells must undergo a reduction or loss of their intracellular junctions. This transition involves the partial or complete acquisition of mesenchymal characteristics, including heightened motility, a loss of apical-basal polarity, and detachment from the basement membrane [57–60]. This crucial mechanism is referred to as Epithelial-to-Mesenchymal Transition (EMT), during which cells exhibit a combination of both epithelial and mesenchymal traits. EMT is recognized as a pivotal method in evolution, particularly during events like gastrulation in embryogenesis [61–64].

EMT represents a multifaceted and multi-stage trans differentiation process through which epithelial cells undertake a series of intricate biochemical alterations to adopt a mesenchymal phenotype. This process holds significant importance, particularly in facilitating the rapid growth and metastatic potential of tumors [65, 66]. It's essential to emphasize that EMT is a reversible mechanism. Besides the structural alterations, molecular-level modifications also wield considerable influence in this process [67]. Throughout this process, epithelial cells undergo a transformation, adopting mesenchymal characteristics, including the expression of markers such as N-cadherin, vimentin, and fibronectin, while concurrently losing their epithelial markers, such as E-cadherin, occludin, claudin, and laminin [68]. The initiation of specific EMT transcriptional pathways is responsible for orchestrating these modifications. Transcriptional regulators such as TWIST, SNAI1, SNAI2, ZEB1, and ZEB2 actively suppress the expression of E-cadherin, while concurrently encouraging the expression of markers associated with mesenchymal differentiation. These markers may include N- and/or R-cadherin, along with vimentin. Additionally, these pathways impact the expression of proteins related to the cellular matrix and focal adhesion, consequently promoting cellular motility [55, 69, 70].

The function of EMT in cancer development is noteworthy and can be understood from various perspectives. Initiation of EMT is associated with the promotion of cancer metastasis. Research findings indicate that glutamine deficiency in PC cells leads to an elevation in the expression of Slug, an upstream mediator of EMT. This upregulation of Slug contributes to the stimulation of the EMT process and, consequently, results in an increased spread of cancer [71]. Cancer cells exert control over the levels of N- and E-cadherin. The expression of N-cadherin is elevated, while E-cadherin levels are reduced, driven by the action of SERPINH1. Consequently, the induction of EMT occurs. Notably, the EMT process instigated by SERPINH1 is reliant on Wnt signaling [72]. β- Catenin is a key element of Wnt signaling and is capable of nuclear translocation, which can influence the expression of targets further down the line.[73]. Upon induction by TXND12, there is a notable nuclear translocation of β-catenin. Subsequently, this nuclear β-catenin activates the upregulation of ZEB1 expression. This process involving β-catenin and ZEB1 plays a pivotal role in instigating EMT-mediated cancer invasion [74].

## Pancreatic cancer stem cell markers as standalone prognostic variables

### Cluster of differentiation 24 (CD24)

CD24 is a relatively compact mucin-like glycosylphosphatidylinositol (GPI)-linked protein present on the cell surface, playing a role in facilitating cell adhesion. This protein is detected in the developmental stages of the kidneys and brain. It seems that CD24 also has relevance in the progress of PC [75]. During the transition from a healthy ductal epithelium to an invasive intraductal papillary mucinous carcinoma, there is a noticeable increase in CD24 expression within the migrating cells. Furthermore, the levels of CD24 expression within intraductal papillary mucinous neoplasms correlate with the degree of atypia, demonstrating an incremental rise in expression in tandem with the severity of atypia [76, 77].

### Cluster of differentiation 133 (CD133)

CD133 is a transmembrane protein localized within lipid rafts. It comprises a cytoplasmic domain capable of tyrosine phosphorylation and an extracellular domain that has the capability to bind gangliosides [78]. The transcription of CD133 is controlled by various transcriptional promoters, including P1, P2, P3, P4, and P5. Several research studies indicate that P5 exerts a significant impact on pancreatic cancer stem cells (PCSCs) by modulating CD133 expression [79]. Extracellular signal-regulated kinases (ERKs), notably ERK1 or ERK2, can start a downstream signaling cascade that will activate the P5 promoter. In response to auto- or paracrine-released nerve growth factor (NGF), the MAPK/ERK pathway is activated through the TrkA receptor, leading to the activation of ERKs within pancreatic cancer stem cells (PCSCs). In contrast, heat shock proteins such as HIF-1 and HIF-2 appear to target the P5 promoter region [80]. An oxygen shortage in the PC zone serves to intensify CD133 transcription, consequently leading to elevated CD133 expression during the process of carcinogenesis within pancreatic tissue [81, 82].

### Doublecortin-like kinase 1 (DCLK1)

A distinct subset of pancreatic cells, displaying notable morphological and functional differences, has been identified in the earliest stages of pancreatic cancer, and these cells have the potential to function as CSCs. These cells are stamped by the presence of the microtubule regulator DCLK1 as a distinguishing marker [83]**.** Upstream regulators of DCLK1 have not undergone a comprehensive investigation as of now [84]**.** The elevated expression of IL17, originating from mesenchymal inflammatory cells and immune cells, potentially contributes to the upregulation of DCLK1 expression. This impact may be brought about by nuclear factor kB (NF-kB) activation via the traditional route, supporting the preservation of stemness in cells [85, 86]. The heightened expression of DCLK1 plays a significant role in regulating numerous intracellular pathways during the malignant transformation of pancreatic tissue, primarily through mechanisms that involve microRNAs [87]. DCLK1 promotes tumorigenesis by upregulating critical drivers of pancreatic tumorigenesis, notably cMyc and KRAS, utilizing a mechanism dependent on the let-7a microRNA [88, 89]**.**

### Cluster of differentiation 44 (CD44)

CD44 is yet another significant marker [90]. Hyaluronic acid (HA), osteopontin, chondroitin, collagen, fibronectin, and serglycin/sulfated proteoglycan serve as membrane receptors for this entity [91]. Indeed, CD44 plays a pivotal role in diverse biological processes, including cellular adhesion, angiogenesis, the release of cytokines, and muscle healing [92]. Evidence points to the possibility that this protein may play a significant role in the development of PC [93]. Twenty exons make up the CD44 gene's coding [94]. The constant form of CD44 is also referred to as the standard CD44 isoform (CD44s), and it comprises exons 1–5 and 16–20 [95]. The variant CD44 isoform (CD44v) encompasses the middle exons, which can be alternatively spliced and combined with the ten exons found in CD44s. As a result, a vast array of CD44 isoforms, potentially numbering in the thousands, can be generated [96].

### C-X-C motif chemokine receptor 4 (CXCR4)

CXCR4 is a G-protein-coupled receptor specifically designed to interact with stromal-derived factor-1 (SDF-1) [97]. While the majority of CXCR4 expression is shown on hematopoietic cells, it may also be seen on stromal cells, endothelial cells, and the surface of mature blood cells [98]. Both CXCR4 and SDF-1 are now recognized to have an increasingly significant function in the pathogenesis of PC [99]. According to Koshiba et al.'s research, CXCR4 expression is often present [100]. In healthy cells, the expression of CXCR4 is under the regulation of Nuclear Respiratory Factor 1 (NRF1) and Ying Yang 1. NRF1 acts as a secondary messenger in numerous cellular signaling pathways, and growth factors stimulate the transcription of CXCR4, while Ying Yang 1 and pro-inflammatory factors exert an opposing effect. CXCR4, upon binding with CXC12, plays a crucial role in facilitating the movement of immune cells inside the bone marrow and lymph nodes [101].

### Octamer-binding transcription factor 4 (Oct4)

Oct4, a member of the POU family of transcription factors, is a crucial regulator responsible for governing differentiation and maintaining pluripotency in cells [102]. The gene encoding the transcription factor Oct4 is located on chromosome 6, specifically on the region 6p21.31 [103]. This protein, Oct4, functions as the key regulator of pluripotency and plays a pivotal role in processes such as cell renewal, reprogramming, and differentiation [104]. Oct4 carries out its regulatory function by binding to a specific octameric sequence motif, precisely ATGCAAAT. This binding capability allows Oct4 to govern gene expression and exert its impact on cellular processes [105]. At the initiation of development, all parent pluripotent cells initially possess this protein, Oct4. However, germinal stem cells exhibit its expression at a later stage during their development [106]. Oct4 exhibits its most robust expression in undifferentiated cells, and its expression levels decrease as cells progress toward a more differentiated state [107]. In conjunction with SOX2 and NANOG, Oct4 exerts influence over the production of various factors, including fibroblast growth factor 4 (FGF4). Additionally, Oct4 contributes to the regulation of genes that play essential roles in cell renewal and differentiation processes [108].

### Mesenchymal-epithelial transition factor (c‑Met)

c-Met is a receptor tyrosine kinase (RTK) encoded by the MET proto-oncogene. Abnormal activation of c-Met triggers an 'invasive growth' program within cancer cells, contributing to their aggressive behavior [109]. The c-Met signaling pathway, initiated by its ligand HGF, engages multiple signaling pathways within tumor cells. These pathways encompass PI3K/Akt, JAK/STAT, Ras/MAPK, and Wnt/β-catenin, collectively contributing to various critical aspects of tumor biology. This includes promoting tumor proliferation, resistance to apoptosis, driving EMT, facilitating angiogenesis, fostering invasion, and enabling metastasis [110]. Numerous solid organ neoplasms, such as PC, demonstrate abnormal activation of the HGF/c-Met axis. This aberrant activation is strongly linked to alterations in the c-Met gene, including overexpression and amplification, further emphasizing its significance in cancer development and progression [111]. The HGF/c-Met axis plays a pivotal role in the intricate interplay between tumor and stromal cells. This axis contributes to various aspects of pancreatic neoplasms, including in vivo resistance in genetically engineered mouse (GEM) models and the metastatic potential of therapy-resistant tumor cells [112]. Evidence has demonstrated the significance of HGF/c-Met signaling in the maintenance of pancreatic progenitor cells and stem cells. This signaling pathway appears to play a crucial role in regulating the cellular dynamics of these cell populations in the pancreas [113].

### Epithelial cellular adhesion molecule (EpCAM)

EpCAM (Epithelial Cell Adhesion Molecule) is a type I epithelial transmembrane glycoprotein that functions as a homophilic cell–cell adhesion molecule, and its adhesive properties are independent of calcium ions (Ca^2+^) [113]. EpCAM is engaged in a diverse range of physiological, developmental, and pathological processes, contributing to a broad functional spectrum [114]. By exerting influence over cell–cell junctions, signaling pathways, cellular proliferation, polarity, and motility, EpCAM plays a pivotal role in preserving the homeostasis of epithelial tissues [115]. Beyond its involvement in developmental processes, a significant proportion of epithelial tumor tissues, including those derived from metastases such as PC, exhibit heightened or newly induced expression of EpCAM [116]. This heightened expression of EpCAM in tumor tissues, including PC, could be linked to its active role in regulating the growth and metabolism of both fibroblasts and epithelial cells. EpCAM's function includes the rapid induction of the proto-oncogene c-Myc, as well as control over essential cell cycle genes, such as cyclin A and E [117, 118].

## Notch pathway as an oncogenic pancreatic pathway

The developmental signalling pathways, such as WNT, Notch, and Hedgehog, commonly employed by normal stem cells, are also utilized by CSCs. In both healthy and pathological circumstances, these common pathways play a crucial role in regulating a number of cellular functions, including as self-renewal, differentiation, and the preservation of stem cell-like properties [119]. Hedgehog, Notch, Wnt, nuclear factor kappa B (NF-κB), and AKT are among the signalling pathways that undergo alterations in PCSCs and cells undergoing EMT. These pathways, particularly Hedgehog, Notch, and Wnt, assume pivotal roles in regulating essential aspects of PCSCs. They are instrumental in governing the self-renewal ability of PCSCs, as well as tumor initiation, invasive properties, metastatic behavior, and their resistance to therapeutic interventions [120]. It is generally acknowledged that by focusing on signalling pathways crucial for the regulation of PCSCs, chemotherapeutic results can be significantly enhanced. Signalling pathways have a significant impact on PCSCs by influencing pancreatic development, tumor formation, invasion, metastasis, and resistance to therapy. Therefore, studying and targeting these pathways is crucial for understanding and addressing PCSC-related issues. Identifying the causal role that these cellular pathways play in the emergence and growth of CSCs will make it easier to create novel therapeutic strategies to combat this debilitating condition.

The Notch signaling pathway governs transcription factors and growth factors, including Snail, Slug, and TGF, to orchestrate critical processes such as cell proliferation, survival, apoptosis, and differentiation across diverse malignancies, encompassing PC cells and PCSCs. Furthermore, Notch signaling plays a supportive role in promoting EMT. This pathway targets a multitude of genes pivotal for the growth and dissemination of human cancers [120]. Several research studies focused on the CSC Notch pathway have consistently shown that the activation of Notch contributes to the enhancement of metastatic processes, self-renewal capabilities, and cell survival, all the while suppressing apoptotic mechanisms [121]. Various tumor types and subtypes exhibit diverse expressions of distinct Notch ligands and receptors, underscoring the dual roles of Notch as both an oncogene and a suppressor gene. Notch shows elevated expression in multiple cancer types, such as pancreatic, breast, colon, and stomach cancer. Conversely, Notch expression is diminished in select breast malignancies, non-small-cell lung cancer, liver cancer, prostate cancer, skin cancer, and other cancer categories. The specific microenvironment influences whether Notch acts as an oncogene or a tumor suppressor gene. Additionally, Notch receptors undergo post-translational changes that affect their intracellular stability and ligand affinities [121].

In addition to its regulatory influence on CSCs and EMT, the Notch pathway exhibits intricate crosstalk with various other signalling pathways that facilitate the proliferation of cancer cells. Notable interactions include Ras, Wnt, NF-B, Janus Kinase/Signal Transducer and Activator of Transcription (JAK/STAT) signalling, and several others [122]. The simultaneous overexpression of Ras and Notch-1 led to the malignancy induction in human mammary epithelial cells (HMLE), while individual overexpression of either gene did not yield such effects. This finding indicates a mutual reliance between these pathways. Furthermore, JAK/STAT and NF-B signalling pathways exhibit bidirectional interactions with the Notch pathway [123]. Given that NF-κB is a Notch target gene [124], Inhibition of the Notch pathway could potentially lead to the inactivation of the NF-κB pathway. When considering these results collectively, it implies that the modulation of Notch activity may influence the functioning of several other pathways associated with tumor proliferation [125].

**Fig. 4:**
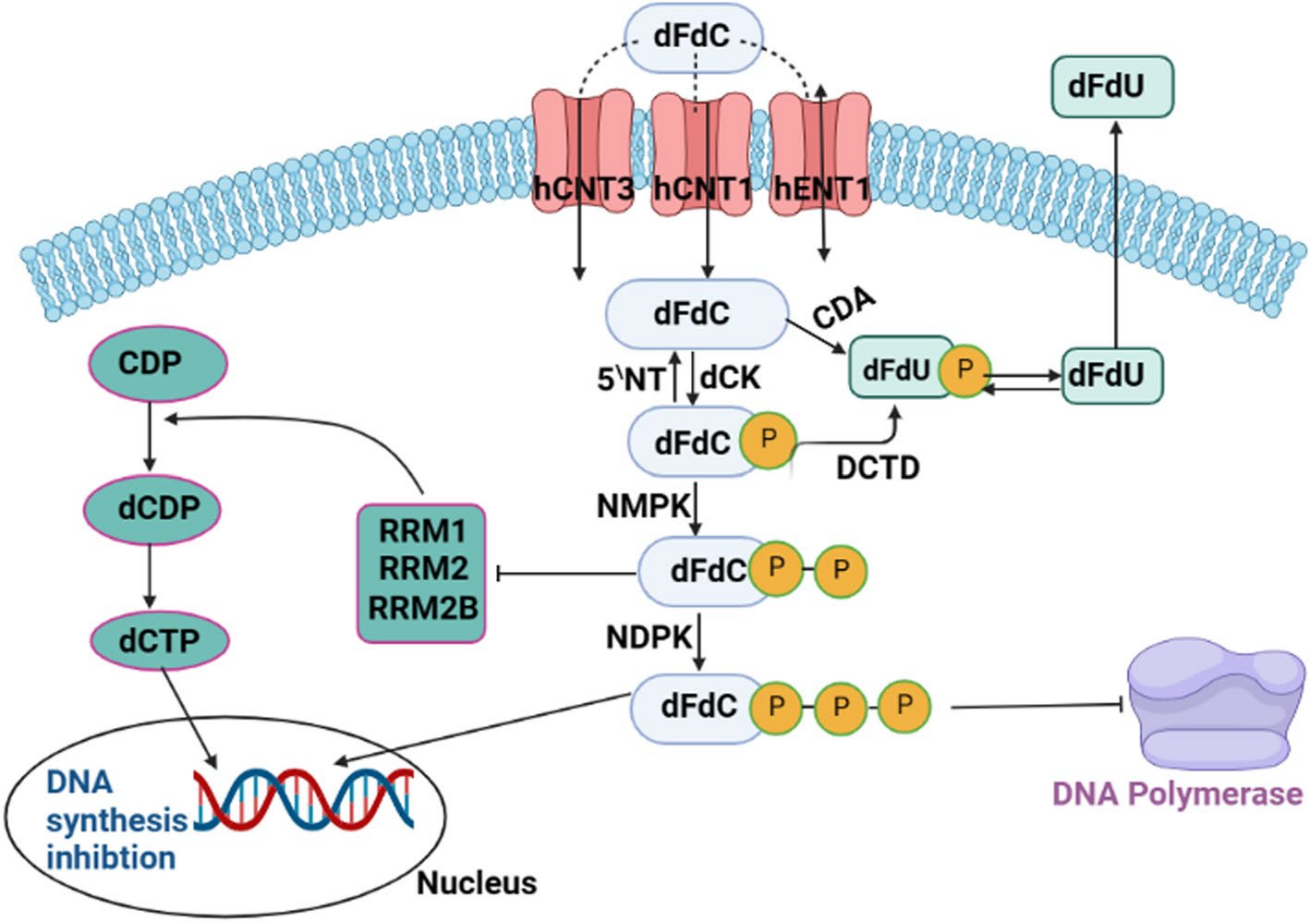
Schematic of the mechanism of action of gemcitabine

**Fig. 5:**
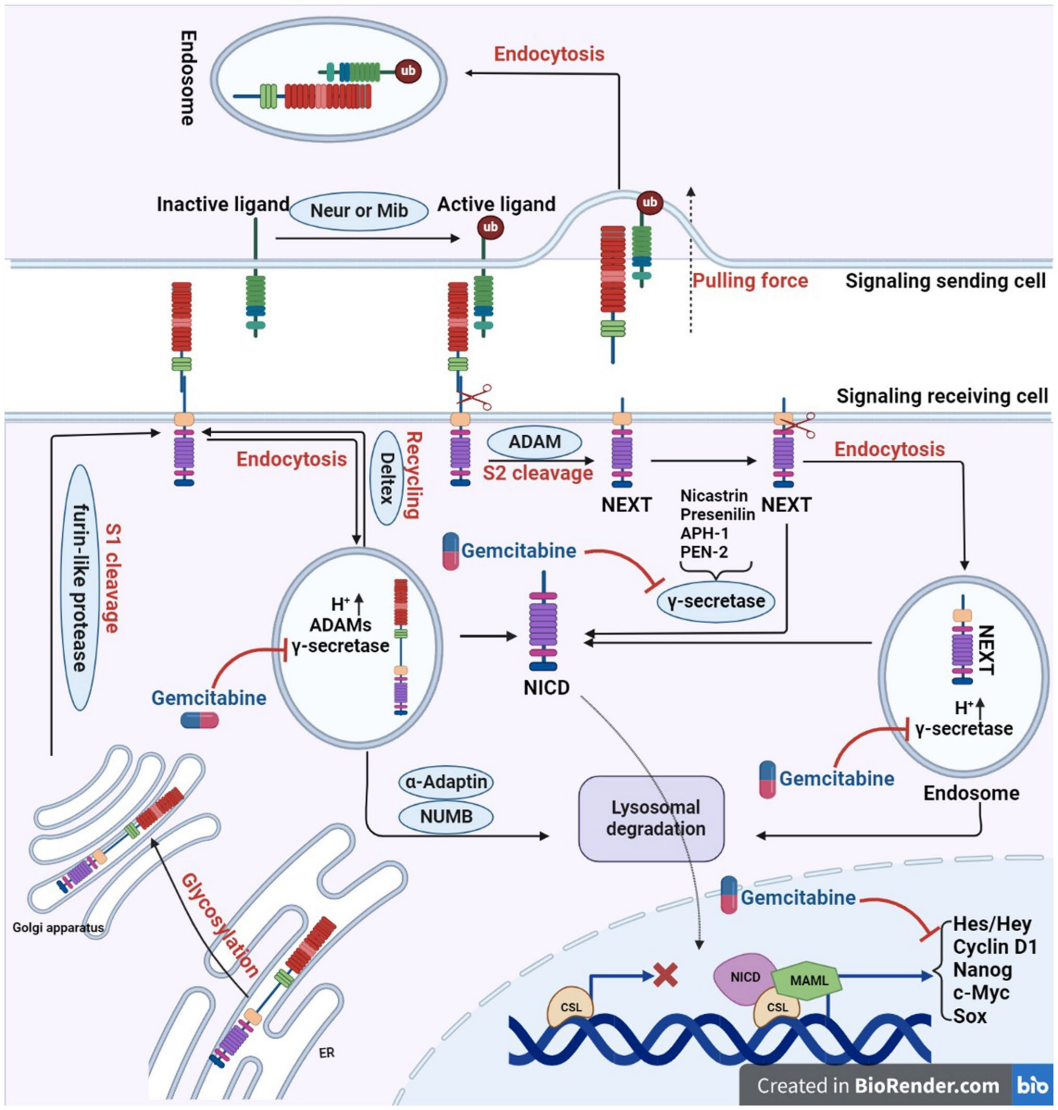
Schematic of Notch pathway and the effect of gemcitabine

The Notch signalling pathway is composed of various components, including the Notch receptor, the Notch ligand (DSL protein), CSL (CBF-1/suppressor of hairless/Lag1), a DNA-binding protein, additional effectors, and various regulatory molecules [121]. Notch signalling is initiated by the direct interaction between Notch ligands and Notch receptors present on the cells that send the signalling as depicted in (Fig. 5 Jagged-1, Jagged-2, and the Delta-like (DLL) family members, notably DLL-1, DLL-3, and DLL-4, are among the five single-pass transmembrane Notch ligands found in mammals [125]. In addition to the extracellular factors involved, certain intracellular genes play a role in regulating the Notch signaling pathway. For example, a membrane-associated protein called MAP17 (also known as DD96 or PDZKIP1) is present in both the plasma membrane and the Golgi apparatus. Notably, MAP17 is not glycosylated. In cervical CSCs, MAP17 interacts with NUMB through its PDZ-binding domain, leading to the activation of the Notch pathway [121]. Nanog regulates Notch signalling along with ALDH [126]. In the human genome, NOTCH1 is located on chromosome 9, NOTCH2 on chromosome 1, NOTCH3 on chromosome 19, and NOTCH4 on chromosome 6. The NOTCH precursor proteins are synthesized within the endoplasmic reticulum (ER) and subsequently transported to the Golgi apparatus after transcription and translation as illustrated in (Fig. 4). During this process, the EGF-like repeat domain of the NOTCH precursors undergoes initial glycosylation within the ER. These glycosylation processes entail the reactions O-fucosylation, O-glucosylation, and O-GlcNAcylation, each of which is catalysed by a different particular enzyme, such as POFUT1, POGLUT1, and EOGT1 [127]. A Furin-like protease catalyses the S1 cleavage of the glycosylated NOTCH precursors in the Golgi apparatus before they are delivered to the cell membrane (Fig. 4) [128]. S1 cleavage splits NOTCH receptors into heterodimers, which are then transported to the cell membrane (Fig. 4). A portion of the NOTCH receptors located on cell membranes undergoes endocytosis into endosomes with the assistance of ubiquitin ligases, leading to a form of ligand-independent activation as depicted in (Fig. 4). Within the acidic environment of endosomes, both a disintegrin and metalloproteinase (ADAMs) and secretase enzymes are present. The NOTCH receptors within endosomes may undergo different fates, which include degradation in lysosomes, recycling back to the cell membrane, or being subjected to cleavage, resulting in the release of the Notch intracellular domain (NICD) (Fig. 4) [128].

The initiation of the Notch signalling pathway requires the activation of the Notch ligand in the signalling-sending cell. This activation is facilitated by the process of ubiquitylation, carried out by the proteins Neuralized (Neur) and Mindbomb (Mib), as illustrated in (Fig. 4). It is noteworthy that in the absence of either Neur or Mib, the NOTCH signalling activity experiences a significant decrease [129, 130], therefore after ubiquitylation, ligands can be endocytosed, thus producing a pulling force for the binding receptors (Fig. 4). The NOTCH receptors are buried in the absence of the pulling force, making them resistant to cleavage by proteins containing the ADAMs domain (S2 cleavage) [128]. Upon S2 cleavage, the residual portion of the NOTCH receptor is referred to as NOTCH extracellular truncation (NEXT), encompassing the transmembrane domain and the intracellular domain. NEXT can undergo further processing by γ-secretase on the cell membrane, leading to the release of Notch intracellular domain (NICD) – this constitutes the ligand-dependent endocytosis-independent activation mechanism. Alternatively, in the ligand-dependent endocytic activation mode, NEXT can be internalized into endosomes, where it can either be cleaved into NICD or directed to lysosomes for degradation. Thus, three distinct approaches contribute to the generation of NICD, categorized as ligand-independent activation, ligand-dependent endocytosis-independent activation, and ligand-dependent endocytic activation, as depicted in (Fig. 4) [128]. Upon translocation into the nucleus, Notch intracellular domain (NICD) interacts with CSL (CBF-1/suppressor of hairless/Lag1) and recruits MAMLs (Mastermind-like proteins), thereby facilitating the transcription of NOTCH target genes, as illustrated in **(**Fig. 4). Numerous strategies exist for inhibiting NOTCH signalling in the context of therapy. One approach involves the design of inhibitors targeting pivotal components of the pathway, such as gemcitabine.

## Gemcitabine

Gemcitabine, also known as 2′,2′-difluoro2′ deoxycytidine or by its trade name Gemzar, is utilized in cancer treatment both as a monotherapy and in combination with other chemotherapeutic agents [131]. Combinations of gemcitabine with other chemotherapeutic agents are sought after due to the potential for additive or synergistic effects, resulting in improved treatment outcomes compared to monotherapy. However, it's important to note that such combination therapies often come with the drawback of increased toxicity, which has led to the preference of using gemcitabine as a standalone treatment in many cases [132].

### Mechanism of action

#### Gemcitabine intracellular metabolism

Gemcitabine's cellular entry is facilitated by nucleoside transporters present on the cell membrane, specifically human equilibrated nucleoside transporters (hENTs) and human concentrative nucleoside transporters (hCNTs). These transporters are essential for gemcitabine, as it has hydrophilic properties, and it needs their assistance to traverse the lipid cell membrane. The intracellular uptake of gemcitabine plays a pivotal role in inhibiting DNA synthesis, which is crucial for its clinical efficacy. Studies in human cell kinetics have indicated that the primary route of intracellular uptake is through hENT1, followed by hENT2, hCNT1, and hCNT3, as illustrated in (Fig. 5) [133, 134].

#### Gemcitabine’s intracellular activation

Once inside the cell, the initial and rate-controlling activation step involves phosphorylation by an enzyme known as deoxycytidine kinase. Subsequently, two more phosphates are added through the action of two enzymes: nucleoside monophosphate kinase and nucleoside diphosphate kinase. This sequential phosphorylation process is essential for gemcitabine's activation within the cellular environment [135]. The culmination of this process leads to the generation of difluorodeoxycytidine triphosphate (dFdCTP) [132]. Gemcitabine halts the cell cycle by impeding DNA synthesis and initiating the apoptotic process [132], and this occurs through.

Gemcitabine is a powerful inhibitor of Ribonucleotide Reductase)RR(, which converts cytidine diphosphate to deoxycytidine diphosphate, leading to the complete loss of one of the two subunits that form RR [136]. Due to the structural similarities of dFdCTP, it competes with deoxycytidine triphosphate (dCTP) as a substrate during DNA synthesis. As a result, during replication, it replaces dCTP by integrating into DNA. As seen in (Fig. 5), this inhibits DNA chain elongation, masks the end of DNA chains, and ultimately culminates in apoptotic cell death [134, 137]. Gemcitabine doesn't have excision-repair susceptibility, so it indirectly induces apoptosis [132].

#### Gemcitabine promotes pancreatic cancer cell stemness and Notch1 activation

Exposing pancreatic cancer cells to a low dose of gemcitabine (1–5 µM) for 24 h, which results in minimal cell killing, induces the expression of stemness-associated molecules, namely Bmi1 and Sox2, along with the cancer stem cell marker CD24. Additionally, this therapy improves the cells' capacity to organize into sphere-like clusters. These clusters show an increased number of cell spheres and larger microsphere size following the gemcitabine treatment. However, pretreatment of pancreatic cancer cells with a 10 µM gamma-secretase inhibitor, tert-Butyl (S)-[(2S)-2-[2-(3,5-difluorophenyl) acetamido] propanamido] phenylacetate DAPT, for 24 h prior to gemcitabine treatment eliminates the expression of NICD1 induced by gemcitabine. Subsequent gemcitabine treatment results in Notch1 inhibition, leading to the impairment of the upregulation of Bmi1, Sox2, and CD24 expression as depicted in (Fig. 5). Additionally, the number and size of these spherical clusters decrease after Notch1 inhibition, and the treatment with gemcitabine increases the migratory and invasive capabilities of PC cells. Notably, the suppression of Notch1 significantly reverses these observed enhancements [138]. Furthermore, the pretreatment with DAPT significantly reversed the chemoresistance induced by gemcitabine. These findings clearly demonstrate that gemcitabine has the effect of promoting stemness in PC cells, which leads to increased migration, invasion, and chemoresistance, and this effect is partially mediated through the activation of the Notch1 pathway [139]. While there have been numerous studies exploring the effects of gemcitabine, there remains a notable gap in research regarding the specific relationship between gemcitabine and the Notch1 signaling pathway [139].

### Side effects of gemcitabine

The efficacy of gemcitabine was enhanced when combined with radiotherapy, leading to improved outcomes in cancer treatment. Although the combination of gemcitabine and radiotherapy may result in higher levels of toxicity, these side effects can generally be managed, making it a viable option in comparison to chemotherapy alone. Notably, the addition of involved-field radiotherapy to gemcitabine therapy is linked to superior overall survival compared to gemcitabine treatment in isolation [140]. Similar to other chemotherapy drugs, gemcitabine is associated with several side effects, including but not limited to neutropenia (reduced white blood cell count), stomatitis (inflammation of the mouth), mucositis (inflammation of the mucous membranes), diarrhea, and emesis (vomiting). These adverse effects are common in chemotherapy regimens and need to be carefully managed to minimize their impact on patients undergoing treatment with gemcitabine [141]. The incorporation of natural compounds, like chrysin, as therapeutic agents, has been observed to result in a reduction of the adverse effects and toxicity commonly associated with chemotherapy. Additionally, these natural products appear to contribute to an enhancement of the tumor cells' sensitivity to chemotherapy, thereby potentially improving the overall efficacy of cancer treatment. However, it is crucial to emphasize the need for further research and clinical investigations to comprehensively assess the safety and effectiveness of such natural products when utilized in conjunction with chemotherapy. Careful monitoring and medical oversight are essential components when integrating these complementary strategies into cancer treatment protocols.

## Chrysin (5,7-dihydroxyflavone)

Chrysin is a naturally-occurring flavone compound that is widely present in various plant extracts, notably in substances like propolis, passion fruit (Passiflora species), and honey [142] Chrysin is extensively utilized as a herbal medicine in many Asian countries and has gained significant attention due to its diverse array of health-promoting properties, including antioxidative, anti-inflammatory, anti-allergic, anti-diabetic, anti-estrogenic, antibacterial, and anti-tumor activities. Its potential as an anticancer agent stands out prominently among its various pharmacological effects [143].

Chrysin, has been discovered to trigger a specific form of cell death called ferroptosis in pancreatic cancer cells. This ferroptosis induction is reliant on the autophagy process and enhances the sensitivity of these cancer cells to the chemotherapy drug gemcitabine. Chrysin achieves this effect by directly binding to carbonyl reductase-1 (CBR1), thereby inhibiting its enzymatic activity at both the molecular and cellular levels. Consequently, the cellular levels of reactive oxygen species (ROS) increase, leading to ROS-dependent autophagy. This autophagy process, in turn, results in the degradation of ferritin heavy polypeptide 1 (FTH1) and an elevation in intracellular free iron levels. Ultimately, this cascade of events contributes to ferroptosis in pancreatic cancer cells. These significant findings suggest that chrysin has the potential to augment the sensitivity of pancreatic cancer cells to gemcitabine by inducing ferroptosis, a phenomenon observed both in vitro and in vivo [144, 145].

### Nuclear factor kappa-light-chain-enhancer of activated B cells (NF-KB)

**Fig. 6:**
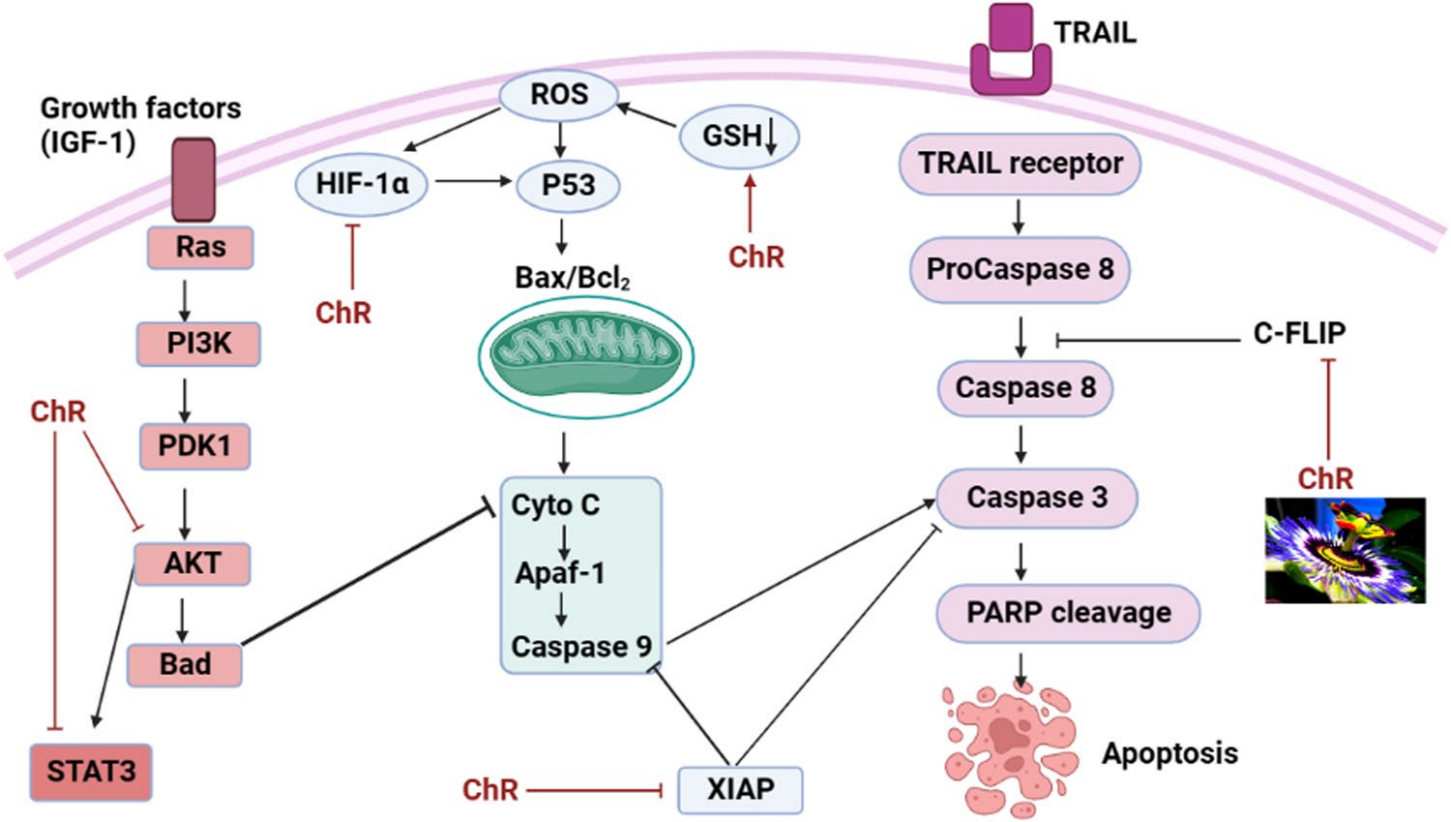
Schematic of the effect of chrysin on NF-KB and Nrf-2 pathways

NF-kB is commonly recognized as a survival issue that enhances the appearance of several anti-apoptotic genes, such as Bcl-2, Bcl-xL, Mcl-1, and c-FLIP (cellular FLICE (FADD-like IL-1b-converting enzyme)-inhibitory protein), which effectively prevent the process of apoptosis [146]. Chrysin, a natural compound, has been observed to enhance the sensitivity of A549 and HeLa human cancer cell lines to TRAIL (Tumor Necrosis Factor-Related Apoptosis-Inducing Ligand) via a specific mechanism. This sensitization effect does not occur by inhibiting TRAIL-induced NF-kB activation or depleting glutathione. Instead, chrysin achieves this effect by downregulating Mcl-1, an anti-apoptotic protein, through the inhibition of STAT3 phosphorylation. This mechanism is supported by the use of cucurbitacin-I, a STAT3-specific inhibitor, which also reduces Mcl-1 levels and increases TRAIL-induced cell death, similar to the effects observed with chrysin treatment as depicted in (Fig. 6) [147].

### Nuclear factor erythroid 2-related factor 2 (Nrf2)

In recent investigations, it has been noted that the activation of the nrf2-mediated signaling pathway is linked to the development of chemoresistance in various cancer cell types. Chrysin has demonstrated the ability to effectively counter chemotherapy resistance associated with nrf2 activation in BEL-7402/ADM cells. This action is achieved by modifying the translocation of Nrf2 into the nucleus, leading to a decrease in the expression of heme oxygenase-1 (HO-1) and NAD(P)H quinone oxidoreductase. Chrysin treatment results in the reduction of nrf2 expression in cells, both at the mRNA and protein levels, through the down-regulation of the PI3K-Akt and ERK signaling pathways [148]. Furthermore, chrysin has been observed to downregulate the protein expression of phosphorylated extracellular signal-regulated kinase 1 and 2 (p-ERK1/2), culminating in its anticancer activity in cancer cell lines through the modulation of the ERK/Nrf2 signaling pathway, as visually depicted in (Fig. 6). [148]

### Limitations of chrysin

Preclinical investigations provide substantiating evidence for the neuroprotective function of chrysin. Nevertheless, clinical studies have been constrained due to the limited bioavailability of the compound [149, 150]. The limited bioavailability of chrysin, measuring at less than 1%, primarily stems from its inadequate solubility in aqueous environments, coupled with its significant pre-systemic and first-pass metabolism [151, 152]. A significant portion of the administered chrysin remains unabsorbed and is subsequently excreted in feces, underscoring the evidence of its notably low bioavailability [151, 153–155]. Due to its limitations, it is carried out as drug delivery as polylactic acid (PLA).

## Polylactic acid (PLA) as drug carrier system

Polylactic acid (PLA) is derived from renewable sources like wheat, straw, corn, and sorghum, making it a type of lactic acid (LA) derivative. Notably, PLA is fully biodegradable [156]. It is friendly to the environment and can break down into water and carbon dioxide [157] and low cost [158]. PLA production involves two forms of lactic acid: L- and D-LA. This process occurs in two stages: first, lactic acid is produced, and then it undergoes either a chemical or biochemical polymerization process to yield PLA [159]. PLA boasts a range of advantageous characteristics, encompassing favorable mechanical properties, thermal stability, ease of processing, thermoplastic behavior, efficient gas barrier, resistance to UV radiation, elasticity, rigidity, hydrophobicity, biocompatibility, and a minimal environmental footprint [160, 161]. The excellent biocompatibility of PLA has made it a prominent choice in the realm of biopharmaceuticals. High molecular weight PLA has been employed for the production of durable surgical sutures, while low molecular weight PLA is utilized to create drug packaging materials with controlled and prolonged drug release properties [162, 163]. PLA's relatively slow decomposition rate and limited cell adhesion capability pose challenges in certain applications, rendering it less water-resistant and less bioactive.

### Pivotal characteristics of PLA

PLA has gained significant prominence in biomedical applications, emerging as a leading material in this domain due to its unique and advantageous characteristics. The primary and pivotal feature of PLA is its remarkable biodegradability, which renders it highly suitable for utilization within DDS. This key attribute ensures that when PLA-based materials enter the body, they can undergo decomposition through natural biological processes. Many DDS rely on the principle of polymer degradation, where the polymer matrix encapsulating the drug gradually corrodes, leading to the controlled release of the therapeutic agent. The biodegradable nature of PLA aligns perfectly with this mechanism, enabling the design of DDS platforms that provide precise and controlled drug release, ultimately enhancing the therapeutic effectiveness while minimizing potential long-term accumulation of the carrier material in the body [164–166]. The process of hydrolysis of PLA leads to the formation of lactic acid monomers. These monomers exhibit a property of not precipitating or accumulating within the body's tissues and living organs. Instead, they can be effectively eliminated through the secretion mechanisms of the kidneys [167]. A significant attribute of PLA is its exceptional biocompatibility and enhanced bioactivity, manifesting as an appropriate response when interacting with the host, as evidenced by its interaction with living tissue [168]. Furthermore, it exhibits controlled absorbency, allowing for continuous drug release, achieved through the regulation of pore size and their interconnectivity [169]. Moreover, its exceptional mechanical attributes facilitate effective medication transportation, mobility, and precise delivery to specific treatment target sites [170]. These attributes have led to its utilization in anti-cancer, anti-inflammatory, anti-diabetic, and antibiotic applications, presenting the potential to mitigate the toxicity and undesired side effects commonly encountered in conventional treatment modalities [171, 172].

### Polylactic-co-glycolic acid (PLGA)

PLGA is a synthetic biodegradable polymer that is widely used for targeted medication delivery and has been licensed by the FDA and the European Association of Medicine [173]. PLGA stands for poly (lactic-co-glycolic acid), which is a copolymer derived from the combination of PLA and poly (glycolic acid) (PGA). Enhancing the biological half-life of PLGA is achieved through a chemical conjugation process with polyethylene glycol (PEG), leading to the creation of nanoparticles (NPs) that envelop its surface. This NP coating acts as a biological shield, employing steric and hydration effects to deter interactions with exogenous molecules. Consequently, the compound's intrabody stability is augmented, culminating in an extended biological half-life attributed to the influence of PEG [174, 175]. The small dimensions of PLGA/PEG molecules, typically within the size range of 50–300 nm, facilitate their penetration through the capillary wall and subsequent dispersion within the capillary structure. This phenomenon contributes to elevated drug accumulation within the designated target region [176]. In an aqueous environment, PLGA/PEG nanoparticles adopt a core–shell architecture. The external shell, endowed with hydrophilic properties, serves to enhance nanoparticle stability. Conversely, the internal core, possessing hydrophobic attributes, provides a suitable platform for the loading of therapeutic agents [177–179]. The incorporation of drug-loaded particles yields enhancements in the pharmacological characteristics of compounds, enabling their preferential accumulation within tumor tissues and cells. This phenomenon is attributed to the augmented permeability and retention effect (EPR), which is leveraged to amplify drug loading while concurrently mitigating undesirable side effects [180, 181]. PLGA/PEG is employed in the formulation of nano-micelles, facilitating the assembly of both hydrophobic and hydrophilic drugs. This orchestrated arrangement enables controlled release of anti-cancer agents, thereby imparting a therapeutic strategy for tumor treatment [182, 183].

### Drug releasing

The drug release mechanism encompasses the elucidation of how drug molecules are liberated and the subsequent characterization of the release rate. PLGA exhibits dynamic motion of its polymer chains and possesses the capacity to imbibe a significant volume of water, resulting in conspicuous swelling. This swelling induces a reorganization of the polymer chain and swollen structure, counteracting the expansion in water content. Consequently, pores are formed, fostering an escalation in diffusion kinetics. The modulation of drug release unfolds through diffusion predominating the early phase, whereas degradation or erosion of the material assumes a pivotal role in dictating release kinetics during the latter phase [184, 185]. Upon introduction of the DDS into an in vitro setting or immersion in an aqueous milieu, the polymer rapidly undergoes water absorption. Within the polymer matrix, the water molecules occupy specific volume regions often denoted as pores, constituting a process characterized as pore formation. During the initial phase, due to the minute dimensions of these pores, only a nominal quantity of drug molecules undergo transport. As time progresses, the water-sustained pores undergo amplification in both size and count, culminating in the formation of an interconnected porous network. This network subsequently facilitates drug release during the later stages. Nevertheless, a heterogeneous degradation event transpires when the PLGA matrix interacts with water, functioning through an auto-catalytic mechanism recognized as hydrolysis. This hydrolytic process instigates the cleavage of ester bonds within the polymer, resulting in a reduction of molecular weight and the generation of acidic byproducts [185, 186].

### Drug targeting

Physiological investigations have revealed substantial disparities between normal and cancerous cells, rendering tumor tissue distinguishable by its compromised blood vessels, attributed to the enlarged pore dimensions of capillaries (ranging from 100 to 780 nm) [187]. Furthermore, the concomitant collapse of lymphatic vessels and the absence of lymphatic circulation within tumor tissues result in the retention of large molecules within the tumor microenvironment. This sequestration occurs due to the hindered ability of the lymphatic system to reabsorb these molecules into the bloodstream. As a consequence, these retained molecules can more readily exert their intended biological effects within the tumor tissue [188]. This effect is called the enhanced EPR [189, 190]. The principle of passive targeting in nanomedicine hinges on this phenomenon, resulting in an extended circulation period within tumor-affected tissues. This effect engenders a discerning drug accumulation specifically within the tumor site, thereby optimizing therapeutic efficacy [191]. Multiple enzymes within the human body participate in the degradation of drugs during the absorption phase, resulting in a diminishment of drug availability in the bloodstream. Nano-scale DDS effectively counteract this phenomenon by enveloping the active compound, thereby shielding it through external physical barriers. This encapsulation strategy serves to enhance drug stability. A pivotal attribute of DDS nanoparticles within the biological milieu is the retention of the encapsulated substance within the bloodstream, obviating the reliance on conventional dosing paradigms seen in alternative drug systems. This pivotal characteristic contributes to an amplification in drug efficiency and efficacy [192]. Nano drug delivery systems play a pivotal role in enhancing drug targeting precision. These systems bring about a controlled alteration in the drug's distribution profile. By utilizing these carriers, the undesirable dispersion of the drug into healthy tissues is curtailed, consequently leading to a reduction in toxicity levels. This mechanism serves to mitigate the potential harm caused by drug leakage into non-targeted areas [177]. In the fabrication of nano DDS, a deliberate adjustment of surface materials is undertaken to tailor their chemical and physical attributes. This strategic modification serves to influence drug loading, pharmacokinetics, and biocompatibility properties. Additionally, it fosters an enhancement in the targeted interaction of these particles with infected cells and tissues, thereby bolstering stability and facilitating sustained drug release.

A noteworthy phenomenon arises due to the rapid proliferation of tumors within the human body. These tumors, due to their aggressive growth, are unable to establish fully developed blood vessels with intact walls. Consequently, a multitude of pores with diameters surpassing that of nano DDS forms. This unique microenvironment enables these DDS to access afflicted tissues and organs, further contributing to their precision in reaching designated pathological sites [192].

### PLA-based delivery of chrysin

While chrysin shows promise as an anti-cancer agent, its poor solubility in water and low bioavailability when taken orally limit its therapeutic potential. Additionally, chrysin gets rapidly metabolized and excreted from the body, making sustained therapeutic levels hard to achieve. PLA offers a solution to the above challenges. PLA is biocompatible, meaning it's safe to use in the human body. It's also biodegradable, breaking down over time into harmless by-products. By encapsulating chrysin in PLA nanoparticles or microspheres, the solubility and stability of chrysin can be significantly improved. This encapsulation ensures controlled and sustained release of chrysin, maintaining therapeutic levels for a more extended period and enhancing its anti-cancer effects [193]. Another way to overcome the challenges of chrysin is load it on PLA. Chrysin-loaded PLA can precisely target pancreatic tumors, minimizing exposure to healthy tissues and reducing potential side effects [194]. Another benefit of using PLA as a carrier is the small size of its nanoparticles which allows better penetration and accumulation within the tumor [193]. This targeted approach ensures that chrysin acts on the tumor cells over an extended period, enhancing its anti-cancer effects and improving the overall therapeutic efficacy against pancreatic tumors [195].

### Limitation of PLA

When constructing clinically significant NPs with several chemotherapeutic drugs, PLA-NPs often have low drug loading despite having high entrapment efficacies. Another issue is the medicines’ sudden release from the NP. The medication may therefore lose its effectiveness if it is unable to get to the target tissue or cells [173]. Drug adsorption on the NP's surface is typically the origin of this initial burst release. In addition, developing techniques for stabilizing acid-sensitive medications is still a hot topic of research because the acid generation that occurs when PLA breaks down is a drawback when adding acid-sensitive medications [196, 197]. To reduce the chance of off-target buildup and adverse consequences, a comprehensive grasp of the pharmacokinetics and pharmacodynamics of both medications and NPs is required. An obstacle for stealth NPs is that they may miss their target and fail to attach to the cell if they are too big. Thus, decreased cellular uptake leads to treatment failure. Further, the commercialization of chemotherapeutic PLA-NPs is also being hampered by issues with reproducibility, scale-up, and related costs [173, 198].

### Suggested strategies about role of chrysin—PLA Nanocomposite in pancreatic cancer stem cells

PLA has improved treatment efficacy in different types of cancer like breast cancer [199, 200], lung cancer [201, 202], prostate cancer [203], Leukemia [204], brain cancer [205], colorectal cancer [206], ovarian cancer [207], liver cancer [208], skin cancer [209]. However, PCSCs is one of the most aggressive and lethal malignancies, often diagnosed at an advanced stage, making it resistant to most treatments. The intricate anatomy of the pancreas, combined with the dense fibrous tissue around pancreatic tumors, makes drug delivery particularly challenging [144, 210]. Chrysin engages in a myriad of different pathways toward pancreatic cancer cells [211]. To embark on, it has been discovered to trigger a specific form of cell death called ferroptosis in PCSCs; different from apoptosis or necrosis, ferroptosis is a controlled type of cell death that depends crucially on iron and lipid peroxidation. Chrysin promotes ferroptosis, which accelerates cancer cell death and increases PCSC susceptibility to gemcitabine, a major chemotherapy for pancreatic cancer. This combination targeting of CBR1 and ferroptosis in it offers a viable treatment approach. Additionally, Chrysin increased cellular ROS levels by variety pathways as recently published and led to ROS-dependent pancreatic cancer cell death, and also demonstrate the effectiveness of p53-Activator pathway regulator, against tumors originating from human PCSCs [211]. Moreover, chrysin downregulated the Nrf2, NF-κB, STAT3, and PI3K/AKT pathways, leading to reduced cancer cell proliferation in PCSCs [144, 212]**.** Furthermore, flavonoids block notch receptor cleavage and/or transcriptional control by the Notch intracellular domain (NICD), flavonoids prevent notch signaling pathway [213]. According to recent studies, notch signaling pathways play a role in cell survival and differentiation, especially in preserving cancer stem cells' ability to self-renew. By lowering the expression of notch1 and its downstream targets, flavonoids like chrysin block this pathway, so compromising the survival and self-renewal of cancer cells [214]. It has been shown that the notch pathway is a key target for flavonoid-based therapies because its inhibition restricts the ability of cancer cells to regenerate directly or indirectly by enhancing sensitivity towards gemcitabine [144]. Moreover, by loading gemcitabine and chrysin onto biodegradable poly lactic acid nanoparticles (NPs), studies have demonstrated that the combination of gemcitabine and flavonoid can play a targeted role and synergistically block the migration of PCSCs because PLA is passively targeting gemcitabine and chrysin of anticancer to the tumor location, and solubilization of hydrophobic pharmaceuticals in their inner core. The harmless PLA copolymer does not build up in the body at low [215]**.** Nevertheless, their well-established preclinical efficacy points out the need for more clinical studies to confirm their therapeutic potential, despite the paucity of clinical trials concentrating on these particular chrysin loaded PLA in PCSCs [216].

## Conclusion

Utilizing appropriate carriers to properly deliver medications to the body is one of the major issues in biomedicine. In this review, we emphasize the understanding of the role of cancer stem cells in pancreatic cancer and the significance of targeting them has paved the way for the development of innovative therapeutic approaches. By targeting the Notch signaling pathway, which plays a crucial role in cancer stem cell self-renewal and differentiation, it is possible to disrupt the tumorigenic properties of pancreatic cancer stem cells and inhibit tumor growth. Additionally, the utilization of polylactic acid-based systems as drug delivery platforms offers advantages such as biocompatibility, controlled drug release, and enhanced bioavailability. Chrysin has shown promise as a chemotherapeutic agent and can contribute to enhancing the efficacy of the treatment approach. Eventually, this review holds the potential to revolutionize pancreatic cancer stem cells treatment by providing targeted therapy to cancer stem cells, improving drug delivery efficiency, and reducing drug resistance. By harnessing the power of nano formulation and understanding the intricate pathways involved in pancreatic cancer stem cells, personalized and effective treatments can be developed that may ultimately improve patient outcomes and survival rates for this devastating disease.

## Data Availability

No datasets were generated or analysed during the current study.

## References

[1] Siegel RL, et al. Cancer statistics, 2023. Ca Cancer J Clin. 2023;73(1):17–48. 36633525 10.3322/caac.21763

[2] Tan P, et al. Artificial intelligence aids in development of nanomedicines for cancer management. Amsterdam: Elsevier; 2023. 10.1016/j.semcancer.2023.01.005 36682438

[3] Siegel RL, et al. Cancer statistics, 2022. CA A Cancer J Clin. 2022;72(1):7–33. 10.3322/caac.21708 35020204

[4] Neoptolemos JP, et al. Therapeutic developments in pancreatic cancer: current and future perspectives. Nat Rev Gastroenterol Hepatol. 2018;15(6):333–48. 29717230 10.1038/s41575-018-0005-x

[5] Ansari D, Gustafsson A, Andersson R. Update on the management of pancreatic cancer: surgery is not enough. World J Gastroenterol: WJG. 2015;21(11):3157. 25805920 10.3748/wjg.v21.i11.3157 PMC4363743

[6] Chen X, et al. Cell death in pancreatic cancer: from pathogenesis to therapy. Nat Rev Gastroenterol Hepatol. 2021;18(11):804–23. 34331036 10.1038/s41575-021-00486-6

[7] Haanen J, Committee EG, et al. Management of toxicities from immunotherapy: ESMO clinical practice guidelines for diagnosis, treatment and follow-up. Ann Oncol. 2017. 10.1093/annonc/mdx225. 28881921 10.1093/annonc/mdx225

[8] Zhao Z, Liu W. Pancreatic cancer: a review of risk factors, diagnosis, and treatment. Technol Cancer Res Treat. 2020;19:1533033820962117. 33357065 10.1177/1533033820962117 PMC7768873

[9] Riquelme E, Maitra A, McAllister F. Immunotherapy for pancreatic cancer: more than just a gut feeling. Cancer Discov. 2018;8(4):386–8. 29610286 10.1158/2159-8290.CD-18-0123

[10] Biankin AV, et al. Pancreatic cancer genomes reveal aberrations in axon guidance pathway genes. Nature. 2012;491(7424):399–405. 23103869 10.1038/nature11547 PMC3530898

[11] Pattanaik SK, et al. A mechanism-based perspective on the use of flavonoids in the treatment of diabetes and its complications. Curr Diabetes Rev. 2024. 10.2174/0115733998335480241022084655.39620338 10.2174/0115733998335480241022084655

[12] Yuan D, et al. Opportunities and challenges in enhancing the bioavailability and bioactivity of dietary flavonoids: a novel delivery system perspective. Food Chem. 2024;430: 137115. 37566979 10.1016/j.foodchem.2023.137115

[13] Helen H, et al. Flavonoids as modulators of miRNA expression in pancreatic cancer: pathways, mechanisms and therapeutic potential. Biomed Pharmacother. 2024;179: 117347. 39241569 10.1016/j.biopha.2024.117347

[14] Khan R, et al. Advances in nanomaterial-based immunosensors for prostate cancer screening. Biomed Pharmacother. 2022;155: 113649. 36108389 10.1016/j.biopha.2022.113649

[15] El-Tanani M, et al. Matrix metalloproteinase 2 is a target of the RAN-GTP pathway and mediates migration, invasion and metastasis in human breast cancer. Life Sci. 2022;310: 121046. 36209829 10.1016/j.lfs.2022.121046

[16] Haggag YA, et al. Nano-encapsulation of a novel anti-Ran-GTPase peptide for blockade of regulator of chromosome condensation 1 (RCC1) function in MDA-MB-231 breast cancer cells. Int J Pharm. 2017;521(1–2):40–53. 28163220 10.1016/j.ijpharm.2017.02.006

[17] Sinha VR, Khosla L. Bioabsorbable polymers for implantable therapeutic systems. Drug Dev Ind Pharm. 1998;24(12):1129–38. 9876570 10.3109/03639049809108572

[18] Basu A, et al. Poly (lactic acid) based hydrogels. Adv Drug Deliv Rev. 2016;107:192–205. 27432797 10.1016/j.addr.2016.07.004

[19] Stryer L, Berg JM, Tymoczko JL. Biochemistry and study guide. WH Freeman: New York NY USA; 2002.

[20] Gullapalli S, Wong MS. Nanotechnology: a guide to nano-objects. Chem Eng Prog. 2011;107(5):28–32.

[21] Lawson JC, Blatch GL, Edkins AL. Cancer stem cells in breast cancer and metastasis. Breast Cancer Res Treat. 2009;118(2):241–54. 19731012 10.1007/s10549-009-0524-9

[22] Keyvani-Ghamsari S, et al. Current understanding of epigenetics mechanism as a novel target in reducing cancer stem cells resistance. Clin Epigenetics. 2021;13(1):1–31. 34051847 10.1186/s13148-021-01107-4 PMC8164819

[23] Han J, et al. Cancer stem cell-targeted bio-imaging and chemotherapeutic perspective. Chem Soc Rev. 2020;49(22):7856–78. 32633291 10.1039/d0cs00379d

[24] Batlle E, Clevers H. Cancer stem cells revisited. Nat Med. 2017;23(10):1124–34. 28985214 10.1038/nm.4409

[25] Shackleton M, et al. Heterogeneity in cancer: cancer stem cells versus clonal evolution. Cell. 2009;138(5):822–9. 19737509 10.1016/j.cell.2009.08.017

[26] Aponte PM, Caicedo A. Stemness in cancer: stem cells, cancer stem cells, and their microenvironment. Stem Cells Int. 2017. 10.1155/2017/5619472. 28473858 10.1155/2017/5619472 PMC5394399

[27] Seoane J. Division hierarchy leads to cell heterogeneity. Nature. 2017;549(7671):164–6. 28854167 10.1038/nature23546

[28] Kuşoğlu A, Avcı ÇB. Cancer stem cells: a brief review of the current status. Gene. 2019;681:80–5. 30268439 10.1016/j.gene.2018.09.052

[29] Nassar D, Blanpain C. Cancer stem cells: basic concepts and therapeutic implications. Annu Rev Pathol. 2016;11:47–76. 27193450 10.1146/annurev-pathol-012615-044438

[30] Huang T, et al. Stem cell programs in cancer initiation, progression, and therapy resistance. Theranostics. 2020;10(19):8721. 32754274 10.7150/thno.41648 PMC7392012

[31] Hemmati HD, et al. Cancerous stem cells can arise from pediatric brain tumors. Proc Natl Acad Sci. 2003;100(25):15178–83. 14645703 10.1073/pnas.2036535100 PMC299944

[32] Singh SK, et al. Identification of a cancer stem cell in human brain tumors. Can Res. 2003;63(18):5821–8. 14522905

[33] Collins AT, et al. Prospective identification of tumorigenic prostate cancer stem cells. Can Res. 2005;65(23):10946–51. 10.1158/0008-5472.CAN-05-2018 16322242

[34] Li C, et al. CD54-NOTCH1 axis controls tumor initiation and cancer stem cell functions in human prostate cancer. Theranostics. 2017;7(1):67. 28042317 10.7150/thno.16752 PMC5196886

[35] O’Brien CA, et al. A human colon cancer cell capable of initiating tumour growth in immunodeficient mice. Nature. 2007;445(7123):106–10. 17122772 10.1038/nature05372

[36] Todaro M, et al. IL-4-mediated drug resistance in colon cancer stem cells. Cell Cycle. 2008;7(3):309–13. 18235245 10.4161/cc.7.3.5389

[37] Li C, et al. Identification of pancreatic cancer stem cells. Can Res. 2007;67(3):1030–7. 10.1158/0008-5472.CAN-06-2030 17283135

[38] Zhang S, et al. Identification and characterization of ovarian cancer-initiating cells from primary human tumors. Can Res. 2008;68(11):4311–20. 10.1158/0008-5472.CAN-08-0364 PMC2553722 18519691

[39] Eramo A, et al. Identification and expansion of the tumorigenic lung cancer stem cell population. Cell Death Differ. 2008;15(3):504–14. 18049477 10.1038/sj.cdd.4402283

[40] Kumar VE, et al. Targeting epigenetic modifiers of tumor plasticity and cancer stem cell behavior. Cells. 2022;11(9):1403. 35563709 10.3390/cells11091403 PMC9102449

[41] Eun K, Ham SW, Kim H. Cancer stem cell heterogeneity: origin and new perspectives on CSC targeting. BMB Rep. 2017;50(3):117. 27998397 10.5483/BMBRep.2017.50.3.222 PMC5422023

[42] Zhao J. Cancer stem cells and chemoresistance: the smartest survives the raid. Pharmacol Ther. 2016;160:145–58. 26899500 10.1016/j.pharmthera.2016.02.008 PMC4808328

[43] Prieto-Vila M, et al. Drug resistance driven by cancer stem cells and their niche. Int J Mol Sci. 2017;18(12):2574. 29194401 10.3390/ijms18122574 PMC5751177

[44] Das PK, Islam F, Lam AK. The roles of cancer stem cells and therapy resistance in colorectal carcinoma. Cells. 2020;9(6):1392. 32503256 10.3390/cells9061392 PMC7348976

[45] Ingangi V, et al. Role of microenvironment on the fate of disseminating cancer stem cells. Front Oncol. 2019;9:82. 30847298 10.3389/fonc.2019.00082 PMC6393337

[46] Lytle NK, Barber AG, Reya T. Stem cell fate in cancer growth, progression and therapy resistance. Nat Rev Cancer. 2018;18(11):669–80. 30228301 10.1038/s41568-018-0056-x PMC8388042

[47] Steinbichler TB, et al. Cancer stem cells and their unique role in metastatic spread. Amsterdam: Elsevier; 2020. 10.1016/j.semcancer.2019.09.007 31521746

[48] Cashman JR, Cashman EA. Effect of PAWI-2 on pancreatic cancer stem cell tumors. Invest New Drugs. 2024. 10.1007/s10637-024-01447-x. 38789849 10.1007/s10637-024-01447-x

[49] Mahadiuzzaman A, Opo FDM, Alkarim S. Stem cell-based targeted therapy in pancreatic cancer: current approaches and future prospects. Tissue Cell. 2024. 10.1016/j.tice.2024.102449. 38924893 10.1016/j.tice.2024.102449

[50] Barman S, et al. Pancreatic cancer and therapy: role and regulation of cancer stem cells. Int J Mol Sci. 2021;22(9):4765. 33946266 10.3390/ijms22094765 PMC8124621

[51] Smith BN, Bhowmick NA. Role of EMT in metastasis and therapy resistance. J Clin Med. 2016;5(2):17. 26828526 10.3390/jcm5020017 PMC4773773

[52] Beuran M, et al. The epithelial to mesenchymal transition in pancreatic cancer: a systematic review. Pancreatology. 2015;15(3):217–25. 25794655 10.1016/j.pan.2015.02.011

[53] Edderkaoui M, et al. An inhibitor of GSK3B and HDACs kills pancreatic cancer cells and slows pancreatic tumor growth and metastasis in mice. Gastroenterology. 2018;155(6):1985–98. 30144430 10.1053/j.gastro.2018.08.028 PMC6328046

[54] Zhou P, et al. The epithelial to mesenchymal transition (EMT) and cancer stem cells: implication for treatment resistance in pancreatic cancer. Mol Cancer. 2017;16(1):1–11. 28245823 10.1186/s12943-017-0624-9 PMC5331747

[55] Rodriguez-Aznar E, et al. EMT and stemness—Key players in pancreatic cancer stem cells. Cancers. 2019;11(8):1136. 31398893 10.3390/cancers11081136 PMC6721598

[56] Pallasch FB, Schumacher U. Angiotensin inhibition, TGF-β and EMT in cancer. Cancers. 2020;12(10):2785. 32998363 10.3390/cancers12102785 PMC7601465

[57] Hwang ST, et al. Corilagin represses epithelial to mesenchymal transition process through modulating Wnt/β-catenin signaling cascade. Biomolecules. 2020;10(10):1406. 33027960 10.3390/biom10101406 PMC7600105

[58] Lee JH, et al. Farnesol abrogates epithelial to mesenchymal transition process through regulating Akt/mTOR pathway. Pharmacol Res. 2019;150: 104504. 31678208 10.1016/j.phrs.2019.104504

[59] Lee JH, et al. Brusatol suppresses STAT3-driven metastasis by downregulating epithelial-mesenchymal transition in hepatocellular carcinoma. J Adv Res. 2020;26:83–94. 33133685 10.1016/j.jare.2020.07.004 PMC7584682

[60] Yang MH, et al. Identification of protocatechuic acid as a novel blocker of epithelial-to-mesenchymal transition in lung tumor cells. Phytother Res. 2021;35(4):1953–66. 33251669 10.1002/ptr.6938

[61] Ashrafizadeh M, et al. MicroRNAs and their influence on the ZEB family: mechanistic aspects and therapeutic applications in cancer therapy. Biomolecules. 2020;10(7):1040. 32664703 10.3390/biom10071040 PMC7407563

[62] Ashrafizadeh M, et al. Role of microRNA/epithelial-to-mesenchymal transition axis in the metastasis of bladder cancer. Biomolecules. 2020;10(8):1159. 32784711 10.3390/biom10081159 PMC7464913

[63] Zañudo JGT, et al. Towards control of cellular decision-making networks in the epithelial-to-mesenchymal transition. Phys Biol. 2019;16(3): 031002. 30654341 10.1088/1478-3975/aaffa1 PMC6405305

[64] Shu DY, Butcher E, Saint-Geniez M. EMT and EndMT: emerging roles in age-related macular degeneration. Int J Mol Sci. 2020;21(12):4271. 32560057 10.3390/ijms21124271 PMC7349630

[65] Lamouille S, Xu J, Derynck R. Molecular mechanisms of epithelial–mesenchymal transition. Nat Rev Mol Cell Biol. 2014;15(3):178–96. 24556840 10.1038/nrm3758 PMC4240281

[66] Rhim AD, et al. EMT and dissemination precede pancreatic tumor formation. Cell. 2012;148(1–2):349–61. 22265420 10.1016/j.cell.2011.11.025 PMC3266542

[67] Kim H, et al. The emerging roles of exosomes as EMT regulators in cancer. Cells. 2020;9(4):861. 32252322 10.3390/cells9040861 PMC7226841

[68] Kalluri R, Weinberg RA. The basics of epithelial-mesenchymal transition. J Clin Investig. 2009;119(6):1420–8. 19487818 10.1172/JCI39104 PMC2689101

[69] Forte E, et al. EMT/MET at the crossroad of stemness, regeneration and oncogenesis: the ying-yang equilibrium recapitulated in cell spheroids. Cancers. 2017;9(8):98. 28758926 10.3390/cancers9080098 PMC5575601

[70] Procacci P, et al. Tumor–stroma cross-talk in human pancreatic ductal adenocarcinoma: a focus on the effect of the extracellular matrix on tumor cell phenotype and invasive potential. Cells. 2018;7(10):158. 30301152 10.3390/cells7100158 PMC6209911

[71] Recouvreux MV, et al. Glutamine depletion regulates Slug to promote EMT and metastasis in pancreatic cancer. J Exp Med. 2020;217:9. 10.1084/jem.20200388 PMC7478719 32510550

[72] Tian S, et al. SERPINH1 regulates EMT and gastric cancer metastasis via the Wnt/β-catenin signaling pathway. Aging (Albany NY). 2020;12(4):3574. 32091407 10.18632/aging.102831 PMC7066881

[73] Ashrafizadeh M, et al. Resveratrol targeting the Wnt signaling pathway: a focus on therapeutic activities. J Cell Physiol. 2020;235(5):4135–45. 31637721 10.1002/jcp.29327

[74] Yuan K, et al. TXNDC12 promotes EMT and metastasis of hepatocellular carcinoma cells via activation of β-catenin. Cell Death Differ. 2020;27(4):1355–68. 31570854 10.1038/s41418-019-0421-7 PMC7206186

[75] Smith SC, et al. The metastasis-associated gene CD24 is regulated by Ral GTPase and is a mediator of cell proliferation and survival in human cancer. Cancer Res. 2006;66(4):1917–22. 16488989 10.1158/0008-5472.CAN-05-3855

[76] Ikenaga N, et al. Characterization of CD24 expression in intraductal papillary mucinous neoplasms and ductal carcinoma of the pancreas. Hum Pathol. 2010;41(10):1466–74. 20619441 10.1016/j.humpath.2010.04.004

[77] Moghadam AR, et al. Ral signaling pathway in health and cancer. Cancer Med. 2017;6(12):2998–3013. 29047224 10.1002/cam4.1105 PMC5727330

[78] Abdalla MY, et al. Enhancing responsiveness of pancreatic cancer cells to gemcitabine treatment under hypoxia by heme oxygenase-1 inhibition. Transl Res. 2019;207:56–69. 30653942 10.1016/j.trsl.2018.12.008

[79] Nagathihalli NS, et al. Inverse correlation of STAT3 and MEK signaling mediates resistance to RAS pathway inhibition in pancreatic cancer. Cancer Res. 2018;78(21):6235–46. 30154150 10.1158/0008-5472.CAN-18-0634 PMC6878978

[80] Xin B, et al. Nerve growth factor regulates CD133 function to promote tumor cell migration and invasion via activating ERK1/2 signaling in pancreatic cancer. Pancreatology. 2016;16(6):1005–14. 27654574 10.1016/j.pan.2016.09.005

[81] Nomura A, et al. NFκB-mediated invasiveness in CD133(+) pancreatic TICs is regulated by autocrine and paracrine activation of IL1 signaling. Mol Cancer Res. 2018;16(1):162–72. 28970361 10.1158/1541-7786.MCR-17-0221 PMC5752573

[82] Chen X, et al. Toll-like receptor 2 and Toll-like receptor 4 exhibit distinct regulation of cancer cell stemness mediated by cell death-induced high-mobility group box 1. EBioMedicine. 2019;40:135–50. 30679086 10.1016/j.ebiom.2018.12.016 PMC6413584

[83] Qiu W, et al. Pancreatic DCLK1(+) cells originate distinctly from PDX1(+) progenitors and contribute to the initiation of intraductal papillary mucinous neoplasm in mice. Cancer Lett. 2018;423:71–9. 29526803 10.1016/j.canlet.2018.03.009 PMC6086584

[84] Zhang Y, et al. Immune cell production of interleukin 17 induces stem cell features of pancreatic intraepithelial neoplasia cells. Gastroenterology. 2018;155(1):210-223.e3. 29604293 10.1053/j.gastro.2018.03.041 PMC6035075

[85] Zhou B, et al. MicroRNA-195 suppresses the progression of pancreatic cancer by targeting DCLK1. Cell Physiol Biochem. 2017;44(5):1867–81. 29224010 10.1159/000485876

[86] Han J, et al. Downregulation of microrna-126 contributes to tumorigenesis of squamous tongue cell carcinoma via targeting KRAS. Med Sci Monit. 2016;22:522–9. 26883054 10.12659/MSM.895306 PMC4760649

[87] Nishio K, et al. Doublecortin and CaM kinase-like-1 as an independent prognostic factor in patients with resected pancreatic carcinoma. World J Gastroenterol. 2017;23(31):5764–72. 28883702 10.3748/wjg.v23.i31.5764 PMC5569291

[88] Ito H, et al. Dominant expression of DCLK1 in human pancreatic cancer stem cells accelerates tumor invasion and metastasis. PLoS ONE. 2016;11(1): e0146564. 26764906 10.1371/journal.pone.0146564 PMC4713149

[89] Li J, et al. Doublecortin-like kinase 1 (DCLK1) regulates B cell-specific moloney murine leukemia virus insertion site 1 (Bmi-1) and is associated with metastasis and prognosis in pancreatic cancer. Cell Physiol Biochem. 2018;51(1):262–77. 30453285 10.1159/000495228

[90] Siegel RL, Miller KD, Jemal A. Cancer statistics, 2019. CA A Cancer J Clin. 2019;69(1):7–34. 10.3322/caac.21551 30620402

[91] Chen C, et al. The biology and role of CD44 in cancer progression: therapeutic implications. J Hematol Oncol. 2018;11(1):64. 29747682 10.1186/s13045-018-0605-5 PMC5946470

[92] Li Z, et al. CD44v/CD44s expression patterns are associated with the survival of pancreatic carcinoma patients. Diagn Pathol. 2014;9:79. 24708709 10.1186/1746-1596-9-79 PMC4108087

[93] Shiina M, Bourguignon LY. Selective activation of cancer stem cells by size-specific hyaluronan in head and neck cancer. Int J Cell Biol. 2015;2015: 989070. 26448762 10.1155/2015/989070 PMC4581563

[94] Hong SP, et al. CD44-positive cells are responsible for gemcitabine resistance in pancreatic cancer cells. Int J Cancer. 2009;125(10):2323–31. 19598259 10.1002/ijc.24573

[95] Yae T, et al. Alternative splicing of CD44 mRNA by ESRP1 enhances lung colonization of metastatic cancer cell. Nat Commun. 2012;3:883. 22673910 10.1038/ncomms1892

[96] Li XP, et al. Expression of CD44 in pancreatic cancer and its significance. Int J Clin Exp Pathol. 2015;8(6):6724–31. 26261555 PMC4525889

[97] Gao Z, et al. Pancreatic stellate cells increase the invasion of human pancreatic cancer cells through the stromal cell-derived factor-1/CXCR4 axis. Pancreatology. 2010;10(2–3):186–93. 20484957 10.1159/000236012

[98] Maréchal R, et al. High expression of CXCR4 may predict poor survival in resected pancreatic adenocarcinoma. Br J Cancer. 2009;100(9):1444–51. 19352387 10.1038/sj.bjc.6605020 PMC2694427

[99] Vandercappellen J, Van Damme J, Struyf S. The role of CXC chemokines and their receptors in cancer. Cancer Lett. 2008;267(2):226–44. 18579287 10.1016/j.canlet.2008.04.050

[100] Cui K, et al. The CXCR4-CXCL12 pathway facilitates the progression of pancreatic cancer via induction of angiogenesis and lymphangiogenesis. J Surg Res. 2011;171(1):143–50. 20462600 10.1016/j.jss.2010.03.001

[101] Rath D, et al. Relative survival potential of platelets is associated with platelet CXCR4/CXCR7 surface exposure and functional recovery following STEMI. Atherosclerosis. 2018;278:269–77. 30342381 10.1016/j.atherosclerosis.2018.10.008

[102] Zaehres H, et al. High-efficiency RNA interference in human embryonic stem cells. Stem Cells. 2005;23(3):299–305. 15749924 10.1634/stemcells.2004-0252

[103] Radzisheuskaya A, Silva JC. Do all roads lead to Oct4? the emerging concepts of induced pluripotency. Trends Cell Biol. 2014;24(5):275–84. 24370212 10.1016/j.tcb.2013.11.010 PMC3976965

[104] Wu G, Schöler HR. Role of Oct4 in the early embryo development. Cell Regeneration. 2014;3(1):7. 25408886 10.1186/2045-9769-3-7 PMC4230828

[105] Zeineddine D, et al. The Oct4 protein: more than a magic stemness marker. Am J Stem Cells. 2014;3(2):74–82. 25232507 PMC4163606

[106] Villodre ES, et al. Roles of OCT4 in tumorigenesis, cancer therapy resistance and prognosis. Cancer Treat Rev. 2016;51:1–9. 27788386 10.1016/j.ctrv.2016.10.003

[107] Lin H, et al. Knockdown of OCT4 suppresses the growth and invasion of pancreatic cancer cells through inhibition of the AKT pathway. Mol Med Rep. 2014;10(3):1335–42. 25017645 10.3892/mmr.2014.2367 PMC4121418

[108] Lu Y, et al. Knockdown of Oct4 and Nanog expression inhibits the stemness of pancreatic cancer cells. Cancer Lett. 2013;340(1):113–23. 23872274 10.1016/j.canlet.2013.07.009

[109] Organ SL, Tsao M-S. An overview of the c-MET signaling pathway. Therapeut Adv Med Oncol. 2011;3(1):7–19. 10.1177/1758834011422556 PMC3225017 22128289

[110] Raj S, et al. Molecular mechanism (s) of regulation (s) of c-MET/HGF signaling in head and neck cancer. Mol Cancer. 2022;21(1):1–16. 35081970 10.1186/s12943-022-01503-1 PMC8790852

[111] Uchikawa E, et al. Structural basis of the activation of c-MET receptor. Nat Commun. 2021;12(1):1–14. 34210960 10.1038/s41467-021-24367-3 PMC8249616

[112] Xu R, et al. c-Met up-regulates the expression of PD-L1 through MAPK/NF-κBp65 pathway. J Mol Med. 2022;100(4):585–98. 35122106 10.1007/s00109-022-02179-2

[113] Choe M, et al. Engaging c-MET (mesenchymal-epithelial transition factor) axis to enhance the safety and antitumor function of HER2-CAR T-cells in sarcoma. Transplantation Cell Therapy. 2023. 10.1016/S2666-6367(23)00360-3.

[114] Barzaman K, et al. Anti-cancer therapeutic strategies based on HGF/MET, EpCAM, and tumor-stromal cross talk. Cancer Cell Int. 2022;22(1):1–20. 35986321 10.1186/s12935-022-02658-z PMC9389806

[115] Keller L, Werner S, Pantel K. Biology and clinical relevance of EpCAM. Cell stress. 2019;3(6):165. 31225512 10.15698/cst2019.06.188 PMC6558934

[116] Park DJ, et al. EpCAM-high liver cancer stem cells resist natural killer cell–mediated cytotoxicity by upregulating CEACAM1. J Immunother Cancer. 2020. 10.1136/jitc-2019-000301. 32221015 10.1136/jitc-2019-000301 PMC7206970

[117] Ruzinova MB, et al. SOX9 expression is superior to other stem cell markers K19 and EpCAM in predicting prognosis in hepatocellular carcinoma. Am J Surg Pathol. 2023;47(1):1–11. 36322988 10.1097/PAS.0000000000001990

[118] Liu Y, et al. Understanding the versatile roles and applications of EpCAM in cancers: from bench to bedside. Exp Hematol Oncol. 2022;11(1):1–19. 36369033 10.1186/s40164-022-00352-4 PMC9650829

[119] Najafi M, Farhood B, Mortezaee K. Cancer stem cells (CSCs) in cancer progression and therapy. J Cell Physiol. 2019;234(6):8381–95. 30417375 10.1002/jcp.27740

[120] Safa AR. Epithelial-mesenchymal transition: a hallmark in pancreatic cancer stem cell migration, metastasis formation, and drug resistance. J Cancer Metast Treat. 2020. 10.20517/2394-4722.2020.55. 10.20517/2394-4722.2020.55 PMC8623975 34841087

[121] Yang L, et al. Targeting cancer stem cell pathways for cancer therapy. Signal Transduct Target Ther. 2020;5(1):1–35. 32296030 10.1038/s41392-020-0110-5 PMC7005297

[122] Rizzo P, et al. Rational targeting of Notch signaling in cancer. Oncogene. 2008;27(38):5124–31. 18758481 10.1038/onc.2008.226

[123] Osipo C, et al. Off the beaten pathway: the complex cross talk between Notch and NF-κB. Lab Invest. 2008;88(1):11–7. 18059366 10.1038/labinvest.3700700

[124] Capaccione KM, Pine SR. The Notch signaling pathway as a mediator of tumor survival. Carcinogenesis. 2013;34(7):1420–30. 23585460 10.1093/carcin/bgt127 PMC3697894

[125] Kiesel VA, Stan SD. Modulation of Notch signaling pathway by bioactive dietary agents. Int J Mol Sci. 2022;23(7):3532. 35408894 10.3390/ijms23073532 PMC8998406

[126] Zhou H-M, et al. Targeting cancer stem cells for reversing therapy resistance: mechanism, signaling, and prospective agents. Signal Transduct Target Ther. 2021;6(1):1–17. 33589595 10.1038/s41392-020-00430-1 PMC7884707

[127] Kovall RA, et al. The canonical Notch signaling pathway: structural and biochemical insights into shape, sugar, and force. Dev Cell. 2017;41(3):228–41. 28486129 10.1016/j.devcel.2017.04.001 PMC5492985

[128] Zhou B, et al. Notch signaling pathway: architecture, disease, and therapeutics. Signal Transduct Target Ther. 2022;7(1):1–33. 35332121 10.1038/s41392-022-00934-y PMC8948217

[129] Daskalaki A, et al. Distinct intracellular motifs of Delta mediate its ubiquitylation and activation by Mindbomb1 and Neuralized. J Cell Biol. 2011;195(6):1017–31. 22162135 10.1083/jcb.201105166 PMC3241720

[130] Wang, W. and G. Struhl, Distinct roles for Mind bomb, Neuralized and Epsin in mediating DSL endocytosis and signaling in Drosophila*.* 2005. 10.1242/dev.01860 15930117

[131] Zhang X-W, et al. Gemcitabine in combination with a second cytotoxic agent in the first-line treatment of locally advanced or metastatic pancreatic cancer: a systematic review and meta-analysis. Target Oncol. 2017;12(3):309–21. 28353074 10.1007/s11523-017-0486-5

[132] Koltai T, et al. Resistance to gemcitabine in pancreatic ductal adenocarcinoma: a physiopathologic and pharmacologic review. Cancers. 2022;14(10):2486. 35626089 10.3390/cancers14102486 PMC9139729

[133] Young JD, et al. The human concentrative and equilibrative nucleoside transporter families, SLC28 and SLC29. Mol Aspects Med. 2013;34(2–3):529–47. 23506887 10.1016/j.mam.2012.05.007

[134] Sarvepalli D, et al. Gemcitabine: a review of chemoresistance in pancreatic cancer. Crit Rev Oncog. 2019. 10.1615/CritRevOncog.2019031641. 31679214 10.1615/CritRevOncog.2019031641

[135] Alvarellos ML, et al. PharmGKB summary: gemcitabine pathway. Pharmacogenet Genomics. 2014;24(11):564. 25162786 10.1097/FPC.0000000000000086 PMC4189987

[136] Smith AB III, et al. Design and synthesis of a tetrahydropyran-based inhibitor of mammalian ribonucleotide reductase. Bioorg Med Chem Lett. 1998;8(22):3133–6. 9873690 10.1016/s0960-894x(98)00575-7

[137] Huang P, et al. Action of 2′, 2′-difluorodeoxycytidine on DNA synthesis. Can Res. 1991;51(22):6110–7. 1718594

[138] Scheel C, Weinberg RA. Cancer stem cells and epithelial–mesenchymal transition: concepts and molecular links. Amsterdam: Elsevier; 2012. 10.1016/j.semcancer.2012.04.001 PMC6220425 22554795

[139] Zhang Z, et al. Hypoxia potentiates gemcitabine-induced stemness in pancreatic cancer cells through AKT/Notch1 signaling. J Exp Clin Cancer Res. 2018;37(1):291. 30486896 10.1186/s13046-018-0972-3 PMC6263055

[140] Loehrer P Sr, et al. A randomized phase III study of gemcitabine in combination with radiation therapy versus gemcitabine alone in patients with localized, unresectable pancreatic cancer: E4201. J Clin Oncol. 2008;26(15):4506–4506.

[141] Hauner K, Maisch P, Retz M. Nebenwirkungen der chemotherapie. Urologe. 2017;56(4):472–9. 10.1007/s00120-017-0338-z 28251254

[142] Altawash ASA, et al. Chrysin-induced sperm parameters and fatty acid profile changes improve reproductive performance of roosters. Theriogenology. 2017;104:72–9. 28822243 10.1016/j.theriogenology.2017.07.022

[143] Kasala ER, et al. Chemopreventive and therapeutic potential of chrysin in cancer: mechanistic perspectives. Toxicol Lett. 2015;233(2):214–25. 25596314 10.1016/j.toxlet.2015.01.008

[144] Zhou L, et al. Chrysin induces autophagy-dependent ferroptosis to increase chemosensitivity to gemcitabine by targeting CBR1 in pancreatic cancer cells. Biochem Pharmacol. 2021;193: 114813. 34673014 10.1016/j.bcp.2021.114813

[145] Zhang Y, Xie J. Induction of ferroptosis by natural phenols: a promising strategy for cancer therapy. Phytother Res. 2024;38(4):2041–76. 38391022 10.1002/ptr.8149

[146] Dreesen O, Brivanlou AH. Signaling pathways in cancer and embryonic stem cells. Stem cell reviews. 2007;3(1):7–17. 17873377 10.1007/s12015-007-0004-8

[147] Lirdprapamongkol K, et al. Chrysin overcomes TRAIL resistance of cancer cells through Mcl-1 downregulation by inhibiting STAT3 phosphorylation. Int J Oncol. 2013;43(1):329–37. 23636231 10.3892/ijo.2013.1926

[148] Gao A-M, et al. Chrysin enhances sensitivity of BEL-7402/ADM cells to doxorubicin by suppressing PI3K/Akt/Nrf2 and ERK/Nrf2 pathway. Chem Biol Interact. 2013;206(1):100–8. 23994249 10.1016/j.cbi.2013.08.008

[149] Balakrishnan R, et al. Natural phytochemicals as novel therapeutic strategies to prevent and treat Parkinson’s disease: current knowledge and future perspectives. Oxidat Med Cell Longevity. 2021. 10.1155/2021/6680935. 10.1155/2021/6680935 PMC8169248 34122727

[150] Gao S, et al. Developing nutritional component chrysin as a therapeutic agent: Bioavailability and pharmacokinetics consideration, and ADME mechanisms. Biomed Pharmacother. 2021;142: 112080. 34449320 10.1016/j.biopha.2021.112080 PMC8653576

[151] Hofer SJ, et al. Caloric restriction mimetics in nutrition and clinical trials. Front Nutr. 2021;8: 717343. 34552954 10.3389/fnut.2021.717343 PMC8450594

[152] Dong D, et al. Sodium oleate-based nanoemulsion enhances oral absorption of chrysin through inhibition of UGT-mediated metabolism. Mol Pharm. 2017;14(9):2864–74. 27983856 10.1021/acs.molpharmaceut.6b00851

[153] Ge S, et al. Determination of pharmacokinetics of chrysin and its conjugates in wild-type FVB and Bcrp1 knockout mice using a validated LC-MS/MS method. J Agric Food Chem. 2015;63(11):2902–10. 25715997 10.1021/jf5056979

[154] Mohos V, et al. Interaction of chrysin and its main conjugated metabolites chrysin-7-sulfate and chrysin-7-glucuronide with serum albumin. Int J Mol Sci. 2018;19(12):4073. 30562928 10.3390/ijms19124073 PMC6320863

[155] Mohos V, et al. Effects of chrysin and its major conjugated metabolites chrysin-7-sulfate and chrysin-7-glucuronide on cytochrome P450 enzymes and on OATP, P-gp, BCRP, and MRP2 transporters. Drug Metab Dispos. 2020;48(10):1064–73. 32661014 10.1124/dmd.120.000085

[156] Singhvi M, et al. d-(−)-Lactic acid production from cellobiose and cellulose by Lactobacillus lactis mutant RM2-2 4. Green Chem. 2010;12(6):1106–9.

[157] Li G, et al. Synthesis and biological application of polylactic acid. Molecules. 2020;25(21):5023. 33138232 10.3390/molecules25215023 PMC7662581

[158] Li J, et al. Poly (lactic acid) controlled drug delivery. Industrial Applications of Poly (lactic acid). 2018;109:138.

[159] de Albuquerque TL, et al. Polylactic acid production from biotechnological routes: a review. Int J Biol Macromol. 2021;186:933–51. 34273343 10.1016/j.ijbiomac.2021.07.074

[160] Farah S, Anderson DG, Langer R. Physical and mechanical properties of PLA, and their functions in widespread applications—a comprehensive review. Adv Drug Deliv Rev. 2016;107:367–92. 27356150 10.1016/j.addr.2016.06.012

[161] Shetty SD, Shetty N. Investigation of mechanical properties and applications of polylactic acids—a review. Mater Res Express. 2019;6(11): 112002.

[162] Li H, Huneault MA. Effect of nucleation and plasticization on the crystallization of poly (lactic acid). Polymer. 2007;48(23):6855–66.

[163] Lampe KJ, et al. Impact of lactic acid on cell proliferation and free radical-induced cell death in monolayer cultures of neural precursor cells. Biotechnol Bioeng. 2009;103(6):1214–23. 19408314 10.1002/bit.22352 PMC2748734

[164] Iqbal N, et al. Recent concepts in biodegradable polymers for tissue engineering paradigms: a critical review. Int Mater Rev. 2019;64(2):91–126.

[165] Guo C, Niu Y. Cellular automaton simulation for degradation of poly lactic acid with acceleratable reaction-diffusion model. ACS Biomater Sci Eng. 2019;5(4):1771–83. 33405553 10.1021/acsbiomaterials.9b00015

[166] Shin DY, et al. In vitro and in vivo evaluation of polylactic acid-based composite with tricalcium phosphate microsphere for enhanced biodegradability and osseointegration. J Biomater Appl. 2018;32(10):1360–70. 29544380 10.1177/0885328218763660

[167] Gutiérrez-Sánchez M, et al. RGD-functionalization of PLA/starch scaffolds obtained by electrospinning and evaluated in vitro for potential bone regeneration. Mater Sci Eng, C. 2019;96:798–806. 10.1016/j.msec.2018.12.003 30606593

[168] Nampoothiri KM, Nair NR, John RP. An overview of the recent developments in polylactide (PLA) research. Biores Technol. 2010;101(22):8493–501. 10.1016/j.biortech.2010.05.092 20630747

[169] Sun J, et al. Nanofiller reinforced biodegradable PLA/PHA composites: current status and future trends. Polymers. 2018;10(5):505. 30966540 10.3390/polym10050505 PMC6415396

[170] Chen DX. Mechanical properties of native tissues and scaffolds. Heidelberg: Springer; 2019.

[171] Arora B, Bhatia R, Attri P. Bionanocomposites: Green materials for a sustainable future. Amsterdam: Elsevier; 2018.

[172] Mariyanats A, et al. Study of the processes of three-dimensional printing of caprolactone copolymers with methylphosphate groups. Philadelphia: IOP Publishing; 2020.

[173] Alvi M, et al. PLGA-based nanoparticles for the treatment of cancer: current strategies and perspectives. AAPS Open. 2022;8(1):12.

[174] Tobio M, et al. Stealth PLA-PEG nanoparticles as protein carriers for nasal administration. Pharm Res. 1998;15(2):270–5. 9523314 10.1023/a:1011922819926

[175] Samkange T, et al. Influence of PEGylation on PLGA nanoparticle properties, hydrophobic drug release and interactions with human serum albumin. J Pharm Pharmacol. 2019;71(10):1497–507. 31385295 10.1111/jphp.13147

[176] Ochekpe NA, Olorunfemi PO, Ngwuluka NC. Nanotechnology and drug delivery part 2: nanostructures for drug delivery. Trop J Pharmaceut Res. 2009;8:3.

[177] Xia W, Chang J. Well-ordered mesoporous bioactive glasses (MBG): a promising bioactive drug delivery system. J Control Release. 2006;110(3):522–30. 16375986 10.1016/j.jconrel.2005.11.002

[178] Xia W, et al. The pH-controlled dual-drug release from mesoporous bioactive glass/polypeptide graft copolymer nanomicelle composites. Eur J Pharm Biopharm. 2008;69(2):546–52. 18248801 10.1016/j.ejpb.2007.11.018

[179] Akbarzadeh A, et al. Preparation and in vitro evaluation of doxorubicin-loaded Fe₃O₄ magnetic nanoparticles modified with biocompatible copolymers. Int J Nanomed. 2012;7:511–26. 10.2147/IJN.S24326 PMC3273983 22334781

[180] Torchilin V. Tumor delivery of macromolecular drugs based on the EPR effect. Adv Drug Deliv Rev. 2011;63(3):131–5. 20304019 10.1016/j.addr.2010.03.011

[181] Lai Y, et al. A novel micelle of coumarin derivative monoend-functionalized PEG for anti-tumor drug delivery: in vitro and in vivo study. J Drug Target. 2012;20(3):246–54. 22118403 10.3109/1061186X.2011.639023

[182] Cao D, et al. Liposomal doxorubicin loaded PLGA-PEG-PLGA based thermogel for sustained local drug delivery for the treatment of breast cancer. Artif Cells Nanomed Biotechnol. 2019;47(1):181–91. 30686051 10.1080/21691401.2018.1548470

[183] Chen W, et al. Synergistic effects of polyethylene glycol and organic montmorillonite on the plasticization and enhancement of poly(lactic acid). J Appl Polym Sci. 2019;136(21):47576.

[184] Fredenberg S, et al. The mechanisms of drug release in poly(lactic-co-glycolic acid)-based drug delivery systems–a review. Int J Pharm. 2011;415(1–2):34–52. 21640806 10.1016/j.ijpharm.2011.05.049

[185] Sharma S, et al. PLGA-based nanoparticles: a new paradigm in biomedical applications. TrAC, Trends Anal Chem. 2016;80:30–40.

[186] Shenderova A, Burke TG, Schwendeman SP. The acidic microclimate in poly(lactide-co-glycolide) microspheres stabilizes camptothecins. Pharm Res. 1999;16(2):241–8. 10100309 10.1023/a:1018876308346

[187] Drummond DC, et al. Optimizing liposomes for delivery of chemotherapeutic agents to solid tumors. Pharmacol Rev. 1999;51(4):691–743. 10581328

[188] García SA, Weitz J, Schölch S. Circulating tumor cells. Methods Mol Biol. 2018;1692:213–9. 28986899 10.1007/978-1-4939-7401-6_18

[189] Lee S, et al. Nano-sized metabolic precursors for heterogeneous tumor-targeting strategy using bioorthogonal click chemistry in vivo. Biomaterials. 2017;148:1–15. 28957709 10.1016/j.biomaterials.2017.09.025

[190] Spitzbarth M, et al. Time-, spectral- and spatially resolved EPR spectroscopy enables simultaneous monitoring of diffusion of different guest molecules in nano-pores. J Magn Reson. 2017;283:45–51. 28881232 10.1016/j.jmr.2017.08.008

[191] Nichols JW, Bae YH. EPR: evidence and fallacy. J Control Release. 2014;190:451–64. 24794900 10.1016/j.jconrel.2014.03.057

[192] Xiao D, Zhou R. Application of nano drug delivery systems in inhibition of tumors and cancer stem cells. Singapore: Springer Singapore; 2021.

[193] Lim HK, et al. Chrysin-induced G protein-coupled estrogen receptor activation suppresses pancreatic cancer. Int J Mol Sci. 2022;23(17):9673. 36077069 10.3390/ijms23179673 PMC9456301

[194] Ashrafizadeh M, et al. A bioinformatics analysis, pre-clinical and clinical conception of autophagy in pancreatic cancer: complexity and simplicity in crosstalk. Pharmacol Res. 2023. 10.1016/j.phrs.2023.106822. 37336429 10.1016/j.phrs.2023.106822

[195] Amirsaadat S, et al. Metformin and Silibinin co-loaded PLGA-PEG nanoparticles for effective combination therapy against human breast cancer cells. J Drug Delivery Sci Technol. 2021;61: 102107.

[196] Fredenberg S, et al. The mechanisms of drug release in poly (lactic-co-glycolic acid)-based drug delivery systems—a review. Int J Pharm. 2011;415(1–2):34–52. 21640806 10.1016/j.ijpharm.2011.05.049

[197] Sunazuka Y, et al. Mechanistic analysis of temperature-dependent curcumin release from Poly (lactic-co-glycolic acid)/Poly (lactic acid) polymer nanoparticles. Mol Pharm. 2024;21(3):1424–35. 38324797 10.1021/acs.molpharmaceut.3c01066

[198] Kumar S, et al. Polymeric (PLGA-based) nanocomposites for application in drug delivery: current state of the art and forthcoming perspectives. Bioresorbable Polymers Composites. 2024;277:324.

[199] Park S-Y, et al. A patient-specific polylactic acid bolus made by a 3D printer for breast cancer radiation therapy. PLoS ONE. 2016;11(12): e0168063. 27930717 10.1371/journal.pone.0168063 PMC5145239

[200] Maji R, et al. Preparation and characterization of Tamoxifen citrate loaded nanoparticles for breast cancer therapy. Int J Nanomed. 2014;3107:3118. 10.2147/IJN.S63535 PMC4077606 25028549

[201] Samadi S, et al. Fabrication of chitosan/poly (lactic acid)/graphene oxide/TiO2 composite nanofibrous scaffolds for sustained delivery of doxorubicin and treatment of lung cancer. Int J Biol Macromol. 2018;110:416–24. 28801095 10.1016/j.ijbiomac.2017.08.048

[202] Zhang K, et al. Cisplatin Polylactic Acid Nanoparticles Combined with miR-181a alleviates the growth, migration and apoptosis of PG cells in lung cancer rats. J Biomed Nanotechnol. 2023;19(6):997–1006.

[203] Ganju A, et al. Nanoways to overcome docetaxel resistance in prostate cancer. Drug Resist Updates. 2014;17(1–2):13–23. 10.1016/j.drup.2014.04.001 PMC4100480 24853766

[204] Tyler B, et al. Polylactic acid (PLA) controlled delivery carriers for biomedical applications. Adv Drug Deliv Rev. 2016;107:163–75. 27426411 10.1016/j.addr.2016.06.018

[205] Shapira-Furman T, et al. Biodegradable wafers releasing Temozolomide and Carmustine for the treatment of brain cancer. J Control Release. 2019;295:93–101. 30605703 10.1016/j.jconrel.2018.12.048

[206] Entezar-Almahdi E, et al. Recent advances in designing 5-fluorouracil delivery systems: a stepping stone in the safe treatment of colorectal cancer. Int J Nanomed. 2020;5445:5458. 10.2147/IJN.S257700 PMC7398750 32801699

[207] Khalifa AM, et al. Current strategies for different paclitaxel-loaded Nano-delivery Systems towards therapeutic applications for ovarian carcinoma: a review article. J Control Release. 2019;311:125–37. 31476342 10.1016/j.jconrel.2019.08.034

[208] Craparo EF, et al. Galactosylated polymeric carriers for liver targeting of sorafenib. Int J Pharm. 2014;466(1–2):172–80. 24607205 10.1016/j.ijpharm.2014.02.047

[209] Hu JK, et al. Nonsurgical treatment of skin cancer with local delivery of bioadhesive nanoparticles. Proc Natl Acad Sci. 2021;118(7): e2020575118. 33526595 10.1073/pnas.2020575118 PMC7896333

[210] Esthar S, et al. An anti-inflammatory controlled nano drug release and pH-responsive poly lactic acid appended magnetic nanosphere for drug delivery applications. Mater Today Commun. 2023;34: 105365.

[211] Shahbaz M, et al. Chrysin a promising anticancer agent: recent perspectives. Int J Food Prop. 2023;26(1):2294–337.

[212] Ragab EM, et al. Impairment of electron transport chain and induction of apoptosis by chrysin nanoparticles targeting succinate-ubiquinone oxidoreductase in pancreatic and lung cancer cells. Genes Nutr. 2023;18(1):1–15. 36906524 10.1186/s12263-023-00723-4 PMC10008604

[213] Singh AK, Kumar S. Flavonoids as emerging notch signaling pathway modulators in cancer. J Asian Nat Prod Res. 2023;1:13. 10.1080/10286020.2023.2202854 37081782

[214] Cai J, et al. Regulation of the Notch signaling pathway by natural products for cancer therapy. J Nutr Biochem. 2024;123: 109483. 37848105 10.1016/j.jnutbio.2023.109483

[215] Serri C, et al. Combination therapy for the treatment of pancreatic cancer through hyaluronic acid-decorated nanoparticles loaded with quercetin and gemcitabine: a preliminary in vitro study. J Cell Physiol. 2019;234(4):4959–69. 30334571 10.1002/jcp.27297

[216] Wan Q, et al. Therapeutic potential of flavonoids from traditional Chinese medicine in pancreatic cancer treatment. Front Nutr. 2024;11:1477140. 39650709 10.3389/fnut.2024.1477140 PMC11620852

